# Meta-analytic estimation of measurement variability and assessment of its impact on decision-making: the case of perioperative haemoglobin concentration monitoring

**DOI:** 10.1186/s12874-016-0107-5

**Published:** 2016-01-19

**Authors:** Emmanuel Charpentier, Vincent Looten, Björn Fahlgren, Alexandre Barna, Loïc Guillevin

**Affiliations:** 1 Secrétariat Scientifique du CEDIT — Assistance Publique - Hôpitaux de Paris, 3, Avenue Victoria, Paris, F-75186 France; 2 CEDIT — Assistance Publique - Hôpitaux de Paris, 3, Avenue Victoria, Paris, F-75186 France

**Keywords:** Methods, meta-analysis as topic, Observer variation, Reproducibility of results, Predictive value of tests, Meta-analysis, Monitoring, intraoperative, Monitoring, physiologic/methods, Biological markers/blood, Hemoglobinometry, Oximetry, **AMS Subject Classification**, Primary 62F15, secondary 92B15

## Abstract

**Background:**

As a part of a larger Health Technology Assessment (HTA), the measurement error of a device used to monitor the hemoglobin concentration of a patient undergoing surgery, as well as its decision consequences, were to be estimated from published data.

**Methods:**

A Bayesian hierarchical model of measurement error, allowing the meta-analytic estimation of both central and dispersion parameters (under the assumption of normality of measurement errors) is proposed and applied to published data; the resulting potential decision errors are deduced from this estimation. The same method is used to assess the impact of an initial calibration.

**Results:**

The posterior distributions are summarized as mean ± sd (credible interval). The fitted model exhibits a modest mean expected error (0.24 ± 0.73 (−1.23 1.59) g/dL) and a large variability (mean absolute expected error 1.18 ± 0.92 (0.05 3.36) g/dL). The initial calibration modifies the bias (−0.20 ± 0.87 (−1.99 1.49) g/dL), but the variability remains almost as large (mean absolute expected error 1.05 ± 0.87 (0.04 3.21) g/dL). This entails a potential decision error (“false positive” or “false negative”) for about one patient out of seven.

**Conclusions:**

The proposed hierarchical model allows the estimation of the variability from published aggregates, and allows the modeling of the consequences of this variability in terms of decision errors. For the device under assessment, these potential decision errors are clinically problematic.

**Electronic supplementary material:**

The online version of this article (doi:10.1186/s12874-016-0107-5) contains supplementary material, which is available to authorized users.

## Background

The CEDIT^1^ is a Health Technology Assessment (HTA) agency within the University Hospitals in Paris (AP-HP^2^). It is in charge since 1982 of advising the senior management about the adoption and use of innovative medical technologies in AP-HP’s hospitals.

We have had to assess, in a limited time frame, the possible impact of the introduction of a device^3^ monitoring the hemoglobin concentration of patients undergoing surgical intervention. This device is used to produce a measurement (SpHb) of the current hemoglobin concentration by means of a sensor which is a variation of the pulse oxymetry sensors; this measure is supposed to replace the measurement (tHb) produced by a laboratory analyzer, thus avoiding the wait for the laboratory results (an element that could be important in a surgical context) and the disruption in the laboratory work flow caused by unplanned requests.

Previous studies of this device in various clinical settings showed that its measurement errors were large but almost symmetric around 0. A recent meta-analysis [1] aggregated the results reported in 32 papers, 13 of which reported results of operating room use; the average mean error (bias) in this surgical subgroup was 0.4 g/dL, but the measurement error standard deviation was larger than 1 g/dL in 15 of the 16 measurement series reported by these 13 papers.

The authors report a bias whose confidence interval includes 0, but they state *“We have not found any publications that provide statistical methods to quantify the uncertainty of SD in meta-analysis”*. Therefore, its clinical conclusions are based on hypotheses on the possible standard deviation of the measurement errors, without estimating it. The authors complete their conclusion on the bias by warning that *“the wide LOA [limits of agreement] mean clinicians should be cautious when making clinical decisions based on these devices”*.

In order to assess the usability of this device, our HTA therefore required the assessment of decision error risks, hence the need to estimate not only the bias (which can be done by a variety of methods, see [2] for an example), but also the variability of the measurements used in this decision. In other words, the use of this device requires not only the assessment of a (possibly “significant”) bias (i.e. an average error whose confidence/credible interval does not contain 0), but also of its variability (e.g. by estimating its standard deviation). This allows us to estimate the probability of a potential clinical decision error.

However, as pointed out by [1], such methods for meta-analytic assessment of variability are almost nonexistent in the field (see Discussion), hence our proposal.

We also wanted to assess the impact of an initial calibration of the device (proposed by some authors in order to remove patient-specific systematic errors) which consists in the subtraction from a given measure SpHb of an initial error SpHb_0_−tHb_0_ obtained from initial calibrating measurements of SpHb and tHb:

$${~}_{c}\text{SpHb}\,=\,\text{SpHb}-\left(\text{SpHb}_{0}-\text{tHb}_{0}\right) $$

Therefore, we propose a Bayesian model allowing to pool the information given in various papers about the distribution of measurement errors, and to use this estimation to assess its impact in the modeling of the clinical decision error risks of these two modes of use of the device.

## Methods

### Literature review

We repeated the published search strategy of [1] on Pubmed and Embase databases, and augmented this search by manual search in the references marked as *“Related to”* by Pubmed; we then obtained full texts of a first selection of papers, whose *“References”* section was used to complete the search. Our selection was driven by the following criteria :

- The device whose operating characteristics were reported in the paper had to use the same operating principle as our target device.
- The paper had to report clinical use during a surgical intervention.
- The paper had to report an estimation of both mean and standard deviation of the differences of paired reference (tHb) and device-derived (SpHb) measurements made at the same time, or at least to quote some indicator (such as Bland & Altman’s LOA [3]) enabling to reconstruct these measures.

The selected papers were analyzed to extract and/or reconstruct sample sizes, observed point estimates of mean and standard deviation of each study population.

### Modeling

For the intended use case (monitoring of hemoglobin concentration in the operating room), the measurement given by reference methods is the only available reference, and the anesthesiologists’ methods are built against this measure. Therefore, we ignored its possible errors and choose to consider tHb, as our standard.

In the selected papers, the same patient may have coupled tHb/SpHb measurements at one or more occasions; we shall see (see Table 1) that in most papers, these different occasions are merged in the same series, without information about intra- and inter-patient variabilities: other papers reported separately measurements made at different occasions, but without information on the possible correlation of measurement errors on the same patient.

**Table 1:** Data extracted from the literature

	Raw SpHb			Calibrated SpHb		
Series	n	m	sd	n	m	sd
Berkow 2011	29.00	−0.30	1.05			
Causey 2011	25.00	−0.30	1.05			
Lamhaut 2011	44.00	0.00	1.40			
Miller 2011	20.00	0.30	1.79			
Applegate 2012	91.00	0.50	1.43			
Butwick 2012-preop	50.00	1.20	1.07			
Butwick 2012-postop	50.00	0.10	1.28			
Butwick 2012-24 h	50.00	1.40	0.99			
Colquhoun 2012	20.00	−1.30	1.94			
Park 2012	40.00	0.90	1.33			
Vos 2012	30.00	−0.20	1.02			
Dewhirst 2013	45.00	−0.10	1.48			
Giraud 2013	53.00	1.00	1.20			
Isosu 2013	92.00	0.20	1.50	71.00	−0.70	1.10
Skelton 2013-preop	137.00	0.60	1.48			
Skelton 2013-postop	137.00	1.60	1.56			
Toyoda 2014-tHbLow	21.00	1.20	1.10			
Toyoda 2014-tHbMed	155.00	−0.20	1.30			
Toyoda 2014-tHbHigh	49.00	−1.00	1.10			
Miyashita 2014-R1-25	71.00	0.60	0.96	71.00	0.15	0.57
Miyashita 2014-R1-25a	73.00	0.68	1.02	73.00	0.16	0.77
Kim 2014-pre	52.00	0.12	1.09			
Kim 2014-Lefort	52.00	0.07	0.94			
Kim 2014-BSSO	52.00	−0.09	0.98			
Kim2014 post	52.00	−0.90	0.85			
Patino 2014	140.00	0.40	1.28	140.00	0.10	1.20
Yamaura 2014-0-1	115.00	0.33	1.41			
Yamaura 2014-1-2	30.00	−0.31	1.24			
Yamaura 2014-2-3	18.00	−0.59	1.11			
Yamaura 2014-3-	12.00	−0.53	0.87			
Saito 2015-Dilution	24.00	1.43	1.24			
Saito 2015-Transfusion	24.00	1.10	1.23			
Awada 2015-Precision	83.00	0.00	0.79			
Frasca 2015	41.00	−0.40	1.40	41.00	−0.30	1.10

n: sample size, m: mean error (SpHb-tHb), sd: standard deviation of error

Therefore, when a paper reported more than one series of measurement errors (i.e. set of assessments of this error made in the same circumstances on independent patients), these series were kept separate, and analyzed as independent: these series were usually characterized by a factor (e.g. operating phase) strongly linked to hemoglobin concentration, overwhelming the (weak) patient-related factors.

In other words, we ignored a possible “paper” level in our model.

#### Raw SpHb

We postulated that in each series *i* in the literature, the individual measurement errors *e*_*i,j,k*_=SpHb_*i,j,k*_−tHb_*i,j,k*_ in patient *j* of the series *i* at occasion *k* are normally distributed (Eq. (1) below). We also postulated that the series-specific means *μ*_*i*_ of measurement errors (i.e. the series-specific biases) are normally distributed in the (hypothetical) population of all possible repetitions of such studies, with a population-level mean *μ*_*m*_ (overall bias) and a population-level standard deviation *σ*_*m*_ (2); similarly, the series-specific standard deviations *σ*_*i*_ are supposed to have a lognormal (*μ*_*s*_,*σ*_*s*_) distribution in the population (3).

(1) $$\begin{array}{*{20}l} \mathrm{e}_{\text{\textit{i,j,k}}} & \sim {\mathcal{N}}\left(\mu_{i}, {\sigma_{i}^{2}}\right)  \end{array} $$

(2) $$\begin{array}{*{20}l} \mu_{i} & \sim {\mathcal{N}}\left(\mu_{m}, {\sigma_{m}^{2}}\right)  \end{array} $$

(3) $$\begin{array}{*{20}l} \sigma_{i} & \sim {\mathcal{L}\mathcal{N}}\left(\mu_{ls}, \sigma_{ls}^{2}\right),~\text{which we shall use as:}  \\ \log\sigma_{i}\ & \sim {\mathcal{N}}\left(\mu_{ls}, \sigma_{ls}^{2}\right)  \end{array} $$

The postulate of normality of measurement errors (1) allows us to use two well-known results of the sampling theory from normal distributions to derive the likelihoods of the usual *m* and *s* estimators of *μ* and *σ* from a sample of size *n*:

(4) $$\begin{array}{*{20}l} \sqrt{n_{i}-1}\,\frac{m_{i}-\mu_{i}}{s_{i}} & \sim t_{n_{i}-1}\qquad\text{and, independently,}  \end{array} $$

(5) $$\begin{array}{*{20}l}[-2pt] (n_{i}-1)\,\frac{{s_{i}^{2}}}{{\sigma_{i}^{2}}} & \sim \chi^{2}_{n_{i}-1}  \end{array} $$

(4) and (5) allow us to compute the likelihoods of the published series-level estimators *m*_*i*_ and *s*_*i*_ instead of requiring patient-level data e_*i,j,k*_.

#### Calibrated SpHb

The error for occasion *k* in patient *j* in series *i*, *e*_*i,j,k*_, is defined by *e*_*i,j,k*_=SpHb_*i,j,k*_−tHb_*i,j,k*_. The error of _*c*_SpHb (“calibrated error”) _*c*_ *e*_*i,j,k*_ will be:

$$\begin{array}{*{20}l} {~}_{c}e_{\text{\textit{i,j,k}}} & = {~}_{c}\text{SpHb}_{\text{\textit{i,j,k}}}-\text{tHb}{i,j,k} \\ {} & = \text{SpHb}_{\text{\textit{i,j,k}}}-\left(\text{SpHb}_{i,j,0}-\text{tHb}_{i,j,0}\right)-\text{tHb}{i,j,k}\\ {} & = \left(\text{SpHb}_{\text{\textit{i,j,k}}}-\text{tHb}{i,j,k}\right) - \left(\text{SpHb}_{i,j,0}-\text{tHb}_{i,j,0}\right)\\ & = e_{\text{\textit{i,j,k}}}-e_{i,j,0}\,. \end{array} $$

Now, in each series *i*, we can decompose *e*_*i,j,k*_ as the sum of a series-specific bias *μ*_*i*_, a patient specific random effect *f*_*i,j*_ distributed with mean 0 and variance ${\tau _{i}^{2}}$, and an occasion-specific random residual *g*_*i,j,k*_ distributed with mean 0 and variance $\upsilon _{i,j}^{2}$.

Suppose further that these terms are independent and, for simplicity, homoscedastic in each series^4^ (i.e. for all patients *j* of the series *i*, $\upsilon _{i,j}^{2}={\upsilon _{i}^{2}}$). Then, $\forall i, \text {Var}\left (e_{\text {\textit {i,j,k}}}\right)={\sigma _{i}^{2}}=\text {Var}\left (\mu _{i}+f_{i,j}+g_{\text {\textit {i,j,k}}}\right)={\tau _{i}^{2}}+{\upsilon _{i}^{2}}$. However,

(6) $$\begin{array}{*{20}l} {~}_{c}e_{\text{\textit{i,j,k}}} & = \mu_{i}+f_{i,j}+g_{\text{\textit{i,j,k}}}-\left(\mu_{i}+f_{i,j}+g_{i,j,0}\right)\\ & = g_{\text{\textit{i,j,k}}}-g_{i,j,0}  \end{array} $$

Therefore, $\text {Var}\left ({~}_{c}e_{\text {\textit {i,j,k}}}\right)=2{\upsilon _{i}^{2}}$. The ratio of corrected to raw measurement standard errors is:

$$\theta_{i}\,= \sqrt{\frac{2{\upsilon_{i}^{2}}}{{\tau_{i}^{2}}+{\upsilon_{i}^{2}}}}\,. $$

Under our assumptions, this ratio can take values between 0 (all error is patient-specific, with no residue, *υ*=0) and $\sqrt {2}$ (all error is random, with no patient-specific component, *τ*=0). Both cases make sense in the current context.

The definition of the calibrated error implies (6) that it is (positively) correlated to the raw error; therefore, their difference should be (negatively) correlated to the raw error, and so should be their means.

It is equivalent to estimate *τ* and *υ* or *σ* and *θ*. The latter allows, as we shall see, to model series with and without calibrated errors in the same way.

We model the impact of calibration as variations of the measurement error’s mean and standard deviation (modeled, as before, as being normally distributed):

(7) $$\begin{array}{*{20}l} {~}_{c}e_{\text{\textit{i,j,k}}} & \sim {\mathcal{N}}\left(\mu\text{\scriptsize c}_{i}, \sigma\text{\scriptsize c}_{i}^{2}\right)  \end{array} $$

(8) $$\begin{array}{*{20}l} \mu\text{\scriptsize c}_{i} & = \mu_{i} + \delta_{i}  \end{array} $$

(9) $$\begin{array}{*{20}l} \sigma\text{\scriptsize c}_{i} & = \sigma_{i}\,\theta_{i}  \end{array} $$

We model the position parameters *μ*_*i*_ and *δ*_*i*_ of individual series as having a bivariate normal distribution; similarly, we model their (suitably transformed) spread parameters *σ*_*i*_ and *θ*_*i*_ as bivariate normally distributed:

(10) $${} {\fontsize{9pt}{9.3pt}\begin{aligned} {\mu_{i} \choose \delta_{i}} \sim\mathcal{MVN} \left({\mu_{m} \choose \mu_{\delta}}, \left({{\sigma_{m}^{2}} \atop \rho_{p}\sigma_{m}\sigma_{\delta}} \ \ \ {\rho_{p}\sigma_{m}\sigma_{\delta} \atop \sigma_{\delta}^{2}} \right)\right)  \end{aligned}}  $$

(11) $${} {\fontsize{9pt}{9.3pt}\begin{aligned} {\log\sigma_{i} \choose \log\frac{\theta_{i}}{\sqrt{2}-\theta_{i}}} \sim\,\mathcal{MVN} \left({\mu_{ls} \choose \mu_{lt}}, \left({\sigma_{lt}^{2} \atop \rho_{s}\sigma_{ls}\sigma_{lt}}\ \ \ {\rho_{s}\sigma_{ls}\sigma_{lt} \atop \sigma_{lt}^{2}} \right)\right)\end{aligned}}  $$

and, as before, (7) allows us to use (4) and (5), *mutatis mutandis,* to compute the likelihoods from the published data.

From (10)–(11) and the properties of the multivariate normal distribution, it follows that the marginal distribution of *μ*_*i*_ is given by (2) and that the marginal distribution of log*σ*_*i*_ is given by (3); therefore, despite the appearances, (2)–(3) describe the same model as (10)–(11) when the calibrated data are unknown.

### Model implementation and fitting

A Bayesian implementation of this model was fitted by MCMC methods, using the Stan [4] modeling language through the rstan [5] interface to R [6]. The model uses Eqs. (4) and (5) to compute the likelihood of the data and directly implements Eqs. (2) and (3) for series without calibrated SpHb and (8) to (11) for series with calibrated SpHb.

Using (1) and (7), we also sampled the relevant parameters of a new study and of a new observation within this study at each iteration of the MCMC sampling, thus obtaining a sample representative of the (predictive) distribution of measurement errors without being constrained by the particulars of any study. This simulation of the characteristics of the device in a new setting is the basis of our inferences on its performance.

Since our data (means and log-standard deviations of errors in the published series) were already more or less centered around 0 and scaled about 1, we followed [7, 8] and choose a Cauchy(0,3) density as a weakly informative prior distribution for the location parameters *μ*_*m*_,*μ*_*δ*_ and the transformed spread parameters *μ*_*ls*_ and *μ*_*lt*_, a half Cauchy(0,3) T[0,] for the standard deviations *σ*_*m*_,*σ*_*δ*_,*σ*_*ls*_ and *σ*_*lt*_, and a Uniform(-1,1) distribution for the correlation coefficients *ρ*_*p*_ and *ρ*_*s*_. This choice allows for a weakly informative prior distribution robust with respect to a few outlier values without expressing unreasonable *a prori* beliefs in very large values of the parameters they model.

The resulting program is available as the Additional file 1; it is also part of the the noweb source of the present paper (see the Additional file 2 for instructions).

The convergence of the MCMC chains was checked by visual assessment of the MCMC traces (see Additional file 3), the ratios of MCMC standard deviation to standard deviation for each parameter of the model (see Additional file 4) and the chain convergence indicator $\widehat {\mathrm {R}}$ (see [9]). The quality of the model was assessed by placing each observed quantity in the *a posteriori* distribution of the parameter it estimates (see Additional file 5).

### Diagnostic impact assessment

We used the bias and standard deviation values created during model parameter estimation to assess the impact of measurement errors in terms of decision errors. We postulated that the true values tHb of hemoglobin concentration were uniformly distributed on the [4 12] g/dL range.

Let *f* the density of the measurement error *E* (whose realizations are the *e*_*i,j,k*_ observations whose mean and standard deviation estimates are reported), and *g* the density of tHb (*F* and *G* being their respective distributions). The probability of observing a measurement SpHb lower than some threshold *t* (a “positive” reading in our case) is:

(12) $$\begin{array}{*{20}l} \Pr(\text{SpHb}<t) & = \Pr(\text{tHb}+E<t)  \\ & = \int_{x}\Pr(x+E<t)\,g(x)\,dx  \\ & = \int_{x}\Pr(E<t-x)\,g(x)\,dx  \\ & = \int_{x}\left(\int_{e<t-x}\!f(e,x)\,de\right)\,g(x)\,d(x)  \\ & = \int_{x}\int_{-\infty}^{t-x}\!f(e,x)\,de\,g(x)\,dx  \end{array} $$

Similarly, the probability of a “true positive” is:

(13) $$\begin{array}{*{20}l} {}\Pr(\text{SpHb}<t\wedge{}\text{tHb}<t) & = \int_{x<t}\int_{-\infty}^{t-x}\!f(e,x)\,de\,g(x)\,dx  \end{array} $$

Since we modeled errors independent of “true” values tHb, these expressions simplify in:

(14) $$\begin{array}{*{20}l} \Pr(\text{SpHb}<t) & = \int_{x} F(t-x)\,g(x)\,dx  \end{array} $$

(15) $$\begin{array}{*{20}l} \Pr(\text{SpHb}<t\wedge{}\text{tHb}<t) & = \int_{-\infty}^{t} F(t-x)\,g(x)\,dx  \end{array} $$

The probability of a “positive” case being *G*(*t*) by definition, (14) and (15) are sufficient to compute the sensitivity, specificity and positive and negative predictive values.

The diagnostic impact of measurement errors depends on the distribution of the true values tHb. For reasons discussed below, we choose to assess this impact by postulating a uniform distribution of tHb on a range spanning the clinically useful range of threshold values. According to the literature, this range is about 6 to 10 g/dL [10–12]. Therefore, our impact assessment used an uniform distribution over the range from 4 to 12 g/dL.

## Results

The literature review led us to select 21 papers [13–33] reporting 34 distinct estimations of the mean and standard deviation of measurement error; among these, four papers [24, 27, 28, 32] report the characteristics of measurement error after initial calibration in five series. The data extracted from the literature are listed in Table 1.

### Model fit

In the text, posterior distributions are summarized as mean ± sd (credible interval) unit; the bounds of the credible intervals are the.025 and.975 quantiles. The full set of summary statistics for the MCMC sample can be found in the Additional file 4.

#### Analysis of raw SpHb measurement errors

**Fig. 1:**
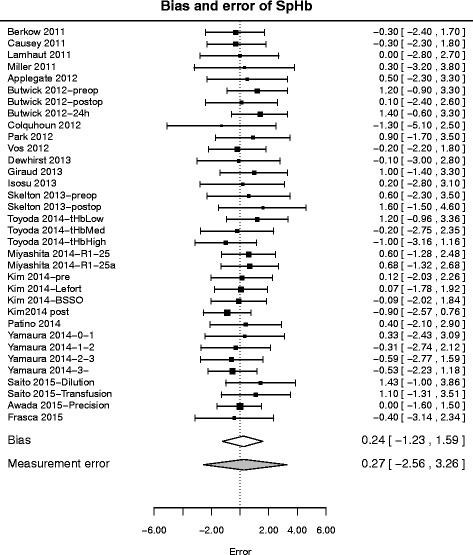
Meta-analysis of raw SpHb errors. Forest plot depicting the data from the literature (*top*) and the inferences made on bias and measurement error by model fitting (*bottom*). Top: square: reported bias (average error) *m*_*i*_ (size proportional to weight), whiskers: possible errors (*m*_*i*_±1.96*s* *d*_*i*_). Bottom: credible regions for bias of a new study (*μ*_∗_) and error of a new observation (*e*_∗,∗,∗_)

The population-level results of the model fitting for raw SpHb measurement errors are depicted in Fig. 1 and summarized in Table 2; Table 3 summarizes predictive error results, i.e. bias and standard deviation in a new study (new setting), and mean error, squared error and absolute error for an new observation.

**Table 2:** Estimates of the population-level distribution of measurement errors of raw SpHb

	Mean	95 % credible interval	
*μ*_*m*_	0.23	−0.02	0.46
*σ*_*m*_	0.71	0.54	0.94
*μ*_*ls*_	0.23	0.15	0.30
*σ*_*ls*_	0.18	0.13	0.26

**Table 3:** Replication simulation results for raw SpHb

	Mean	95 % credible interval	
*μ*_∗_	0.24	−1.23	1.59
*σ*_∗_	1.28	0.86	1.82
*e*_∗,∗,∗_	0.27	−2.56	3.26
$e_{*,*,*}^{2}$	2.24	0.00	11.31
|*e*_∗,∗,∗_|	1.18	0.05	3.36

Distributional characteristics of the expected bias (*μ*_∗_) and standard deviation (*σ*_∗_) of error for a replicated study, and error (*e*_∗,∗,∗_), squared error ($e_{*,*,*}^{2}$) and absolute error (|*e*_∗,∗,∗_|) for a replicated observation

The overall mean error (bias) of raw SpHb has mean 0.23 ± 0.12 (−0.02 0.46) g/dL; the measurement error of raw SpHb is distributed around this mean with log-standard deviation 0.23 ± 0.04 (0.15 0.30) g/dL.

The mean expected bias (systematic error expected in a new study) is 0.24 ± 0.73 (−1.23 1.59) g/dL. The mean expected error (new measurement error in a new study) is 0.27 ± 1.47 (−2.56 3.26) g/dL, whereas the mean expected absolute error is 1.18 ± 0.92 (0.05 3.36) g/dL, and the root of the mean quadratic expected error is 1.50 g/dL.

#### Impact of calibration

**Fig. 2:**
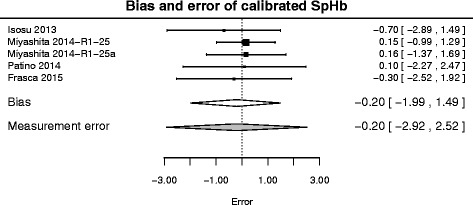
Meta-analysis of calibrated SpHb errors. See Fig. 1 for legend

The population-level estimates of the impact of calibration are presented in Table 4 and Fig. 2 and the simulation-based estimates of the resulting measurement errors are presented in Table 5, which also reports the expected bias correction and expected ratio of raw and calibrated standard deviations (inflation/deflation factor).

**Table 4:** Estimates of the population-level distribution of corrections to measurement error allowed by calibration

	Mean	95 % credible interval	
*μ*_*δ*_	−0.42	−0.83	0.02
*σ*_*δ*_	0.39	0.03	1.28
*ρ*_*p*_	−0.13	−0.92	0.92
*μ*_*lt*_	0.19	−0.33	0.73
*σ*_*lt*_	0.51	0.09	1.43
*ρ*_*ls*_	0.39	−0.52	0.97

**Table 5:** Replication simulation results for calibrated SpHb

	Mean	95 % credible interval	
*μ*_*c*∗_	−0.20	−1.99	1.49
*σ*_*c*∗_	1.00	0.39	1.81
*δ*_∗_	−0.44	−1.55	0.70
*θ*_∗_	0.77	0.36	1.17
*e*_*c*∗,∗,∗_	−0.20	−2.92	2.52
$e_{c*,*,*}^{2}$	1.86	0.00	10.29
|*e*_*c*∗,∗,∗_|	1.05	0.04	3.21

Distributional characteristics of the expected bias (*μ*_*c*_∗), standard deviation of error (*σ*_*c*_∗), correction term (*δ*_∗_) and inflation factor (*θ*∗) for a replicated study, and error (*e*_*c*∗,∗,∗_), squared error ($e_{c*,*,*}^{2}$) and absolute error (|*e*_*c*∗,∗,∗_|)for a replicated observation

One notes that, whereas the bias correction is almost systematically negative (−0.42 ± 0.20 (−0.83 0.02) g/dL), the impact of calibration on standard error and expected errors is modest (the mean expected absolute error is 1.05 ± 0.87 (0.04 3.21) g/dL, which is not much less than in the non-calibrated case), and has a non-negligible probability of enlarging the standard error (actually, for a new study, Pr(*θ*>1)≈ 0.102).

### Estimation of clinical impact

**Fig. 3:**
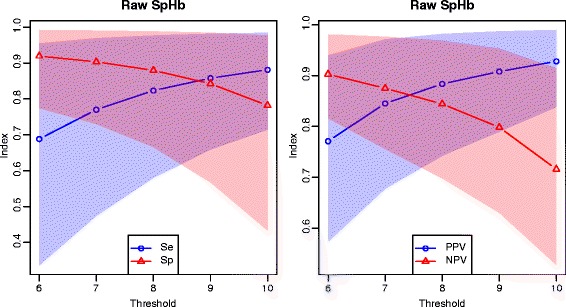
Diagnostic values of raw SpHb. Mean values and 95 % credible intervals; left: sensitivity, specificity; right: predictive values

**Fig. 4:**
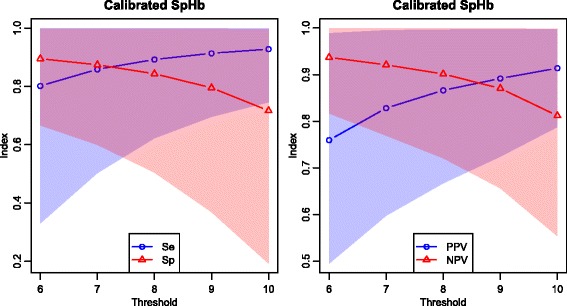
Diagnostic values of calibrated SpHb. Mean values and 95 % credible intervals; left: sensitivity, specificity; right: predictive values

**Fig. 5:**
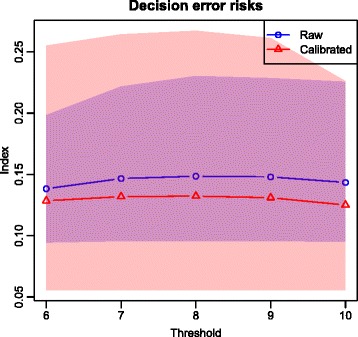
Decision error risks. Mean values and 95 % credible intervals

The decisional impact of measurement errors of raw SpHb is summarized in Table 6 in terms of sensitivity, specificity, positive and negative predictive values (conditional probabilities) as well as accuracy and probability of a decision error (absolute probabilities); these results are illustrated in Fig. 3. Similarly, the Table 7 and the Fig. 4 summarize the diagnostic impact of measurement errors of calibrated SpHb. The resultant risks of decision errors and their credible regions are graphically compared in Fig. 5.

**Table 6:** Clinical impact of measurement errors of raw SpHb

Index	Threshold	Mean	SD	2.5 %	25 %	50 %	75 %	97.5 %
Se	6	0.688	0.168	0.337	0.578	0.710	0.818	0.955
Se	7	0.770	0.134	0.474	0.686	0.791	0.873	0.969
Se	8	0.823	0.106	0.580	0.758	0.842	0.905	0.977
Se	9	0.858	0.086	0.659	0.805	0.873	0.924	0.981
Se	10	0.881	0.072	0.715	0.838	0.894	0.936	0.985
Sp	6	0.919	0.058	0.775	0.889	0.932	0.963	0.992
Sp	7	0.903	0.070	0.731	0.868	0.919	0.956	0.991
Sp	8	0.880	0.087	0.666	0.835	0.899	0.945	0.988
Sp	9	0.842	0.111	0.567	0.783	0.866	0.927	0.985
Sp	10	0.782	0.145	0.433	0.699	0.809	0.893	0.977
PPV	6	0.771	0.096	0.574	0.705	0.776	0.842	0.940
PPV	7	0.845	0.078	0.678	0.795	0.854	0.904	0.971
PPV	8	0.884	0.064	0.743	0.843	0.892	0.933	0.982
PPV	9	0.908	0.053	0.790	0.875	0.916	0.948	0.987
PPV	10	0.928	0.040	0.839	0.902	0.933	0.959	0.990
NPV	6	0.903	0.044	0.817	0.872	0.905	0.936	0.981
NPV	7	0.875	0.059	0.756	0.834	0.880	0.919	0.977
NPV	8	0.844	0.071	0.698	0.793	0.850	0.898	0.969
NPV	9	0.798	0.085	0.630	0.737	0.803	0.860	0.953
NPV	10	0.716	0.101	0.527	0.642	0.716	0.786	0.914
Acc	6	0.862	0.027	0.802	0.847	0.864	0.879	0.906
Acc	7	0.853	0.032	0.778	0.836	0.857	0.875	0.904
Acc	8	0.851	0.034	0.770	0.833	0.856	0.875	0.904
Acc	9	0.852	0.034	0.771	0.834	0.857	0.875	0.904
Acc	10	0.857	0.033	0.774	0.840	0.862	0.878	0.905
Err	6	0.138	0.027	0.094	0.121	0.136	0.153	0.198
Err	7	0.147	0.032	0.096	0.125	0.143	0.164	0.222
Err	8	0.149	0.034	0.096	0.125	0.144	0.167	0.230
Err	9	0.148	0.034	0.096	0.125	0.143	0.166	0.229
Err	10	0.143	0.033	0.095	0.122	0.138	0.160	0.226

Threshold in g/dL; Se: sensitivity, Sp: specificity, PPV: positive predictive value, NPV: negative predictive value, Acc: accuracy, Err: probability of decisional error

**Table 7:** Clinical impact of measurement errors of calibrated SpHb

Index	Threshold	Mean	SD	2.5 %	25 %	50 %	75 %	97.5 %
Se	6	0.801	0.177	0.330	0.712	0.845	0.938	1.000
Se	7	0.858	0.135	0.503	0.795	0.894	0.958	1.000
Se	8	0.892	0.105	0.622	0.845	0.920	0.969	1.000
Se	9	0.913	0.085	0.696	0.876	0.936	0.975	1.000
Se	10	0.928	0.071	0.746	0.896	0.947	0.979	1.000
Sp	6	0.895	0.089	0.666	0.854	0.914	0.958	0.999
Sp	7	0.874	0.106	0.600	0.824	0.897	0.950	0.998
Sp	8	0.843	0.129	0.504	0.781	0.872	0.937	0.998
Sp	9	0.795	0.164	0.369	0.713	0.831	0.916	0.997
Sp	10	0.717	0.208	0.192	0.599	0.754	0.876	0.996
PPV	6	0.760	0.128	0.495	0.672	0.764	0.853	0.989
PPV	7	0.828	0.104	0.598	0.760	0.837	0.907	0.995
PPV	8	0.866	0.086	0.667	0.812	0.876	0.931	0.997
PPV	9	0.892	0.071	0.724	0.847	0.900	0.946	0.998
PPV	10	0.914	0.055	0.788	0.878	0.920	0.956	0.998
NPV	6	0.937	0.049	0.817	0.906	0.946	0.977	1.000
NPV	7	0.921	0.063	0.769	0.884	0.934	0.971	1.000
NPV	8	0.901	0.077	0.721	0.855	0.916	0.963	1.000
NPV	9	0.871	0.094	0.656	0.812	0.886	0.947	0.999
NPV	10	0.812	0.121	0.554	0.731	0.822	0.909	0.998
Acc	6	0.871	0.052	0.745	0.849	0.880	0.904	0.944
Acc	7	0.868	0.054	0.736	0.843	0.878	0.903	0.944
Acc	8	0.868	0.054	0.733	0.843	0.878	0.903	0.944
Acc	9	0.869	0.051	0.739	0.844	0.878	0.903	0.944
Acc	10	0.875	0.044	0.774	0.853	0.881	0.904	0.944
Err	6	0.129	0.052	0.056	0.096	0.120	0.151	0.255
Err	7	0.132	0.054	0.056	0.097	0.122	0.157	0.264
Err	8	0.132	0.054	0.056	0.097	0.122	0.157	0.267
Err	9	0.131	0.051	0.056	0.097	0.122	0.156	0.261
Err	10	0.125	0.044	0.056	0.096	0.119	0.147	0.226

Threshold in g/dL; Se: sensitivity, Sp: specificity, PPV: positive predictive value, NPV: negative predictive value, Acc: accuracy, Err: probability of decisional error

## Discussion

### Methods

#### Modeling

##### Study-level modeling

A mixed-model meta-analysis requires the estimation of one study-level parameter per data point, plus any population-level parameters necessary to the model (in our case, population level mean and standard deviation or, in the case of _*c*_SpHb, differences with SpHb means and ratios to the SpHb standard deviation). This is true both for frequentist, ML-based, estimation and for Bayesian model fitting. Therefore, published meta-analyses usually do not allow for checking of their assumptions on which estimations and inferences are based on the sole basis of published data.

In our case, Eqs. (4) and (5) are crucial. The former is uncontroversial: this result is known to be asymptotically true for any sample of independent and identically distributed observation sampled from a distribution for which the central limit theorem holds; its rate of convergence is known to be good enough for almost any “large” sample (one finds often *N*>30 in common practice in applied statistics), and often considered sufficient for “reasonably” distributed small samples.

The latter is valid only for i.i.d. samples of normally distributed variables. We are not aware of any general asymptotic results concerning the estimation of variability parameters. This scarceness of general results, already noted by [1] has also motivated a recent paper by Nakagawa et al. [34], where the authors build tools for meta-analytic estimation of variability; the relevant tool for their question being the Coefficient of Variation, and ratios thereof, they derive the relevant estimators and their properties.

Their work is based on an unbiased estimator of the log of the standard deviation *σ*:

$$\widehat{\log{\sigma}}\,=\,\log{s}+\frac{1}{2(n-1)} $$

This equality can be derived from the left-hand side of (5). The authors add: *It is assumed that with a large sample size and sufficiently large value of* *σ*, log*σ* *is normally distributed with variance* $s^{2}_{\log \sigma }$. They support this assumption by referencing a 1987 paper by Raudenbush & Bryk, which indeed derive a large-sample theory for this case ([35], pp 250–1). Unfortunately, this paper also states that *“First, the underlying data must be assumed normally distributed, an assumption which can be checked by conventional methods”*. (*ibid.*, p. 252).

In other words, the validity of (5) depends on the accuracy and rate of convergence of log*s*− log*σ* to a normal. We are not aware of any analytic or simulation convergence results for this quantity, but the convergence rate of a ${\chi ^{2}_{n}}$ distribution to a normal is known to be slow.

Since the individual data are unavailable by hypothesis in a meta-analytic context, the normality of the distribution of these data cannot be “checked”. The rate of asymptotic convergence to normality being unknown, the assumption of normality of individual data is a strong necessity of validity of our modeling.

We are not aware of any other literature pertinent to the meta-analytic estimtion of variability.

##### Population-level modeling

The modeling of means (Eq. 2) is the *de facto* standard in meta-analysis. The modeling of standard deviations (Eq. 3) is less so:

By analogy with the sampling distribution of the variance of identically sized normal samples, a gamma distribution was a “natural” candidate for this modeling. However, the interpretation of its parameters was delicate, and the elicitation of priors to these parameters even more so. Therefore, we choose to use a lognormal model of the standard deviations population. The point of this choice was to get a parametrization allowing easy interpretation and easy prior elicitation.

We also modeled *μ*_*i*_ independent of *σ*_*i*_; this assumption simplifies programming, and appears reasonable: in the original data, the correlation of biases and standard deviations is 0.013 (similarly, the correlation of mean _*c*_SpHb and their standard deviations is −0.53, with only 3 d.f.).

##### Modeling of calibrated SpHb

The rationale for modeling _*c*_SpHb as we did has been exposed *supra*. We could have also used a single model, using only (10) and (11) and treating the (hypothetical) values of _*c*_SpHb in studies not reporting it as missing data (supplementary parameters to the model). The results should be equivalent, but the programming would have been more awkward.

##### Prior distributions

We needed to give our hyperparameters a proper prior distribution, in order to get proper posterior distributions and to be able to use the log-posterior samples to estimate a Bayes factor. However, we had very little information on the distribution of our subject of interest before reading the relevant papers; therefore, we choose to use weakly informative priors. Centering them on 0 was uncontroversial. The difficulty was in the choice of shape and scale.

It has been noted that the common ${\mathcal {N}}(0,V^{2})$ for some very large standard deviation *V*, often used as a “weakly informative” prior distribution, expresses a prior belief of absolute values larger than *V* of about 0.32. Choosing an unreasonably high value of *V* is hardly defensible in face of the subject matter.

Our choice of priors was remotely inspired by the work of Gelman et al. [7, 8] and we borrowed their proposed functional form, except for correlation coefficients where a Uniform(−1,1) was a natural choice.

#### Clinical impact assessment

We choose to report the clinical performance of the device by the (absolute) probability of a (potential) decision error; this index seemed more clinically intepretable and useable than specificities and sensitivities (which can be traded off against one another by the choice of threshold), which are conditional probabilities.

Postulating the independence of values of measured variable and measurement error allowed us to use (14) and (15), which can be simply computed, at least with our choice of population distribution of the variable, with a standard numerical integration routine.

They can even be explicitly solved in some cases: for example, a normal density of values and a normal density of errors convolve to a normal density, which can be trivially used to compute the probability of errors. However, this model would not have reasonable medical support in our case.

Without this postulate, the multiple integrals (12) and (13) are much more problematic to compute numerically, and a better solution would probably be to estimate them by one form or another of Monte Carlo integration.

We had also to choose a range of “useful” values to assess the potential clinical impact of the measurement errors.

- It is obvious that real values quite far from the decision threshold do not contribute to false positive/negative (the probability of a large error is small).
- On the other hand, a small region quite close to the decision threshold overstates the risk of false results. For example, with a symmetric error density, given a threshold *T* in a study *i* with average bias *μ*_*i*_, the rate of false negative a test region (*T*+*μ*_*i*_+*ε* *T*+*μ*_*i*_+2*ε*) would have a limit of $\frac {1}{2}$ for *ε*→0^+^.
- Similarly, a strong mode would overstate the importance the weight of the region surrounding that mode.

The anesthesiological literature shows that a “reasonable” region for transfusion decision threshold is [6 10] g/dL; the choice of a threshold in this range for a given patient depends on various domain- and patient-specific factors.

It was therefore necessary to cover this range (with extension to “likely” regions), without justification to choose a mode. This led us to choose the [4 12] g/dL.

A better choice would have been to model the distribution of tHb of measurements done for clinical reasons (i.e. excluding the systematic or calibration measurements). The source papers, however, did not document this information in any usable form.

### Results

#### Raw SpHb

The posterior distribution of measurement errors in a new study is slightly asymmetric around 0 and gives a non-negligible probability to large measurement errors; one also notes that the mean absolute expected error is a large fraction of the range of clinically useful range of measurement.

#### Impact of calibration

One notes that the convergence of the estimation of _*c*_SpHb-related parameters by MCMC is more difficult than for raw SpHb-related ones (smaller n_eff): this might be accounted for by the very low number of available data; only five series after calibration have been published, which might be the absolute minimal sample size for estimating variability.

One also notes that the mean calibrated measurement error is negative. This might have a natural explanation: the calibration is made at the beginning of an intervention, when SpHb is, in general, normal for the patient, whereas the clinically useful measurements are done during intervention, when SpHb might have been lowered by surgical hemorrhage and subsequent perfusion. Several authors [17, 20, 21, 23, 26] have reported, with various levels of precision, a relation between (true) hemoglobin concentration and measurement error; this might explain why the correction, computed on a high-hemoglobin concentration basis, is insufficient to cancel the actual bias observed in low-hemoglobin concentration conditions.

One notes, however, that none of these papers reporting this value-error relationship gave enough detail to allow modeling; our analysis, which is therefore compelled to ignore it, is therefore a simplification of the real situation (but probably not an oversimplification).

#### Use of trends

Several authors have reported to have used SpHb (or SpHb _c_) in terms of trends in time, allowing them to assess the need for a reference measurement tHb rather than the need for transfusion; other authors suggested using these trends, but without reporting actual use. However, these reports were not precise and consistent enough to allow a modeling of this use without resorting to correspondence with the original authors for crucial details. Our time limits precluded such a research.

#### Clinical impact

The probability of a “false report” (false positive or false negative) varies slightly over the range of clinically useful thresholds; however, this probability (13–14 % for raw SpHb, around 13 % for calibrated SpHb) remains clinically problematic: it would affect about one patient out of seven. However, the risk of “effective clinical decision error” is probably lower: the hemoglobin concentration is but one input in a complex decision process, whose analysis on the basis of published information is impossible.

The asymmetry of the diagnostic value curves around the midpoint of the clinically useful range (Figs. 3 and 4) is a consequence of the slight biases of raw SpHb and calibrated SpHb.

One should note (see Fig. 5) that the risks of “false reports” are much more uncertain for calibrated SpHb than for raw SpHb, a consequence (again) of the very low number of published studies on calibrated SpHb.

### Study limitations

The present study has a number of restrictions that limit its significance: Literature review The allocated time for our review precluded an extensive search for gray literature; it also precluded querying the original authors for precisions about their results. Limiting a meta-analysis to formally published data is known to reinforce imprecision.

Similarly, we did not conduct a formal bias risk assessment; this, however, was of lesser consequence for our goals. Study design We did not compare the proposed monitor and the reference method (laboratory measurement) to a common (hypothetical) “gold standard”; instead, we assessed the impact of *substituting* the monitoring to the reference method in terms of clinical decision differences. Since the reference method is the current “clinical gold standard”, it is supposed perfect for clinical purposes, and its possible false positives and false negatives are *ignored*.

Such a comparison, which might have been be worthy if the proposed monitoring had a variability close to the variability of the reference method (various sources quote a mean absolute expected error in the 0.1–0.3 g/dL range), would need an assessment of the reference method, unavailable from published data. Modeling We didn’t even consider fitting a so-called “fixed effects” model, considering that heterogeneity of published data was self-evident.

Lack of time precluded a sensitivity study of the impact of the shape of the study-level parameters distribution and most notably of the shape of the population-level parameters distribution. Our choices seem “reasonable”, but their impact has not been assessed. Further work should assess these impacts.

Similarly, the impact of a departure from the assumption of normality of measurement errors should be systematically assessed, both analytically and with simulation approaches.

Our goals in modeling were limited to the assessment of measurement error and its consequences in terms of decisional errors. In contrast, the authors of [1] created a multiple regression model allowing them to assess the impact of various covariates. This modeling, probably very interesting to anesthesiologists and physiologists, was out of our scope of assessing the practical usability of the device under examination.

Finally, we did not try to assess the reality of the impact of calibration in terms of “hypothesis testing” or “model comparison”: this question was not in our scope of interest. Clinical impact estimation Modeling the clinical consequences of “false reports” is a much more intricate problem, requiring the modelization of a large body of medical knowledge. This question was widely out of the limited scope of the present study.

It should be noted that the main result in terms of clinical impact is an absolute probability of error rather than a conditional probability (such as sensitivity, specificity or predictive values).

## Conclusions

This study has shown that:

- Under the assumption of normality, a hierarchical model of variability can be built and used to estimate the variability of a phenomenon from published aggregate data, without recourse to individual data.
- This estimation can be used to assess the decisional (binary) consequences of the variability of the phenomenon of interest.
- The device of interest has been shown to have a mean absolute expected error of 1.18 ± 0.92 (0.05 3.36) g/dL, which is large when compared to the clinically useful range of measurements.
- The mean measurement error (bias) is 0.24 ± 0.73 (−1.23 1.59) g/dL, whose 95 % credible interval contains 0, and which is negligible compared to the mean absolute expected error.
- This measurement error entails a risk of decision error potentially impacting one patient out of seven, which is clinically problematic.
- This risk of “false report” is therefore much less a consequence of the mean expected error (bias) than a consequence of the mean absolute error (variability of the measurement).
- A calibration of the device using an initial reference measurement does not change this situation to any clinically relevant extent.

The proposed model, whose range of validity remains to be assessed, allows estimation of the variability-bound decision errors risk of a measurement from published aggregates; in the motivating example of hemoglobin concentration monitoring, this estimation shows that its clinical use is problematic.

## Ethical approval and consent

The present paper illustrates the proposed model with an example using already-published data. The authors did not check the conformance of the original papers to the Declaration of Helsinki and relied on the original papers publishers’ checks.

## Standards of reporting

The present paper illustrates the proposed model with an example using already-published data; however, it does not aim to be a full-fledged systematic review of the motivating case. In consequence, the authors did not use the PRISMA checklist; this is discussed as one of the study limitations.

## Availability of supporting data

The data set supporting the results of this article is included within the article as Table 1 and its Additional file 6, noweb source of the article.

## Endnotes

^1^ Comité d’Évaluation et de Diffusion des Innovations Technologiques de l’AP-HP.

^2^ Assistance Publique — Hôpitaux de Paris.

^3^ Masimo Radical-7, Masimo Corp., USA. This device uses an extension of plethysmography by evaluating skin reflectance at 12 different wavelengths.

^4^ The assumption of homoscedasticity of the residual errors allows for a simpler expression of the decomposition of the total error, but is not a necessary condition of validity; the assumption of independence is more crucial.

## Additional files

Additional file 1 **Stan implementation of the meta-analytic model.** (STAN 7.66 kb)

Additional file 2 **(Unix) text file: how to reproduce this paper (incl.software requirements).** (TXT 1.70 kb)

Additional file 3 **MCMC traces (post-warmup).** (PDF 2170.88 kb)

Additional file 4 **Summary of the posterior distribution of all parameters in the model.** (PDF 41.3 kb)

Additional file 5 **Boxplots of study-level parameters distributions against the observed data they model.** (PDF 27.2 kb)

Additional file 6 **noweb (knitr) source of this paper.** Includes data, R and Stan code, bibliographic database. (RNW 182 kb)

## Abbreviations

HTA: Health Technology Assessment: *“the systematic evaluation of the properties and effects of a health technology, addressing the direct and intended effects of this technology, as well as its indirect and unintended consequences, and aimed mainly at informing decision making regarding health technologies”* [36]
tHb: Hemoglobin concentration as measured by the reference method
SpHb: Hemoglobin concentration as measured by the device of interest
_c_SpHb: Hemoglobin concentration as measured by the device of interest and corrected of initial bias
LOA: Limits of agreement, a term widely used in methods comparison studies. See [3]
${\mathcal {N}}(\mu, \sigma ^{2})$: Denotes a normal density of mean *μ* and variance *σ* ^2^
MCMC: Monte-Carlo Markov Chains
ML: Maximum Likelihood
${\mathcal {L}N}(\mu, \sigma ^{2})$: Denotes a lognormal density of location parameter *μ* and spread parameter *σ* ^2^
${\mathcal {M}VN}(\boldsymbol {\mu },\boldsymbol {\Sigma })$: Denotes a multivariate normal density of mean vector ***μ*** and variance-covariance matrix ***Σ***
*t*_*n*_: Denotes a standard Student’s *t* density with *n* degrees of freedom
${\chi ^{2}_{n}}$: Denotes a chi-squared density with *n* degrees of freedom
