# Supplementary figures and images for: Meta-analytic estimation of measurement variability and assessment of its impact on decision-making: the case of perioperative haemoglobin concentration monitoring
Source: BMC Med Res Methodol. 2016 Jan 19;16:7. doi: 10.1186/s12874-016-0107-5 (PMC4717612; doi:10.1186/s12874-016-0107-5)

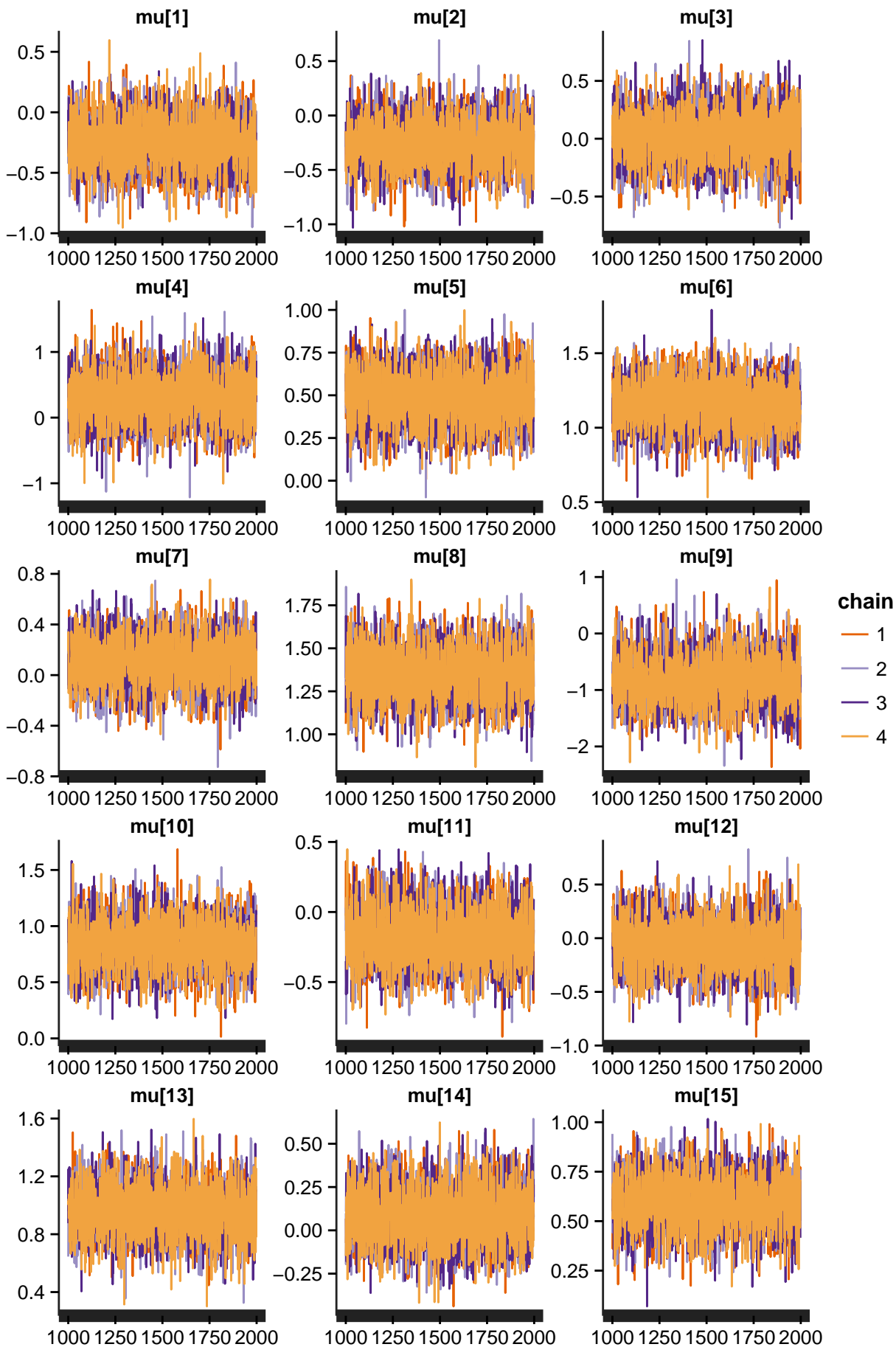

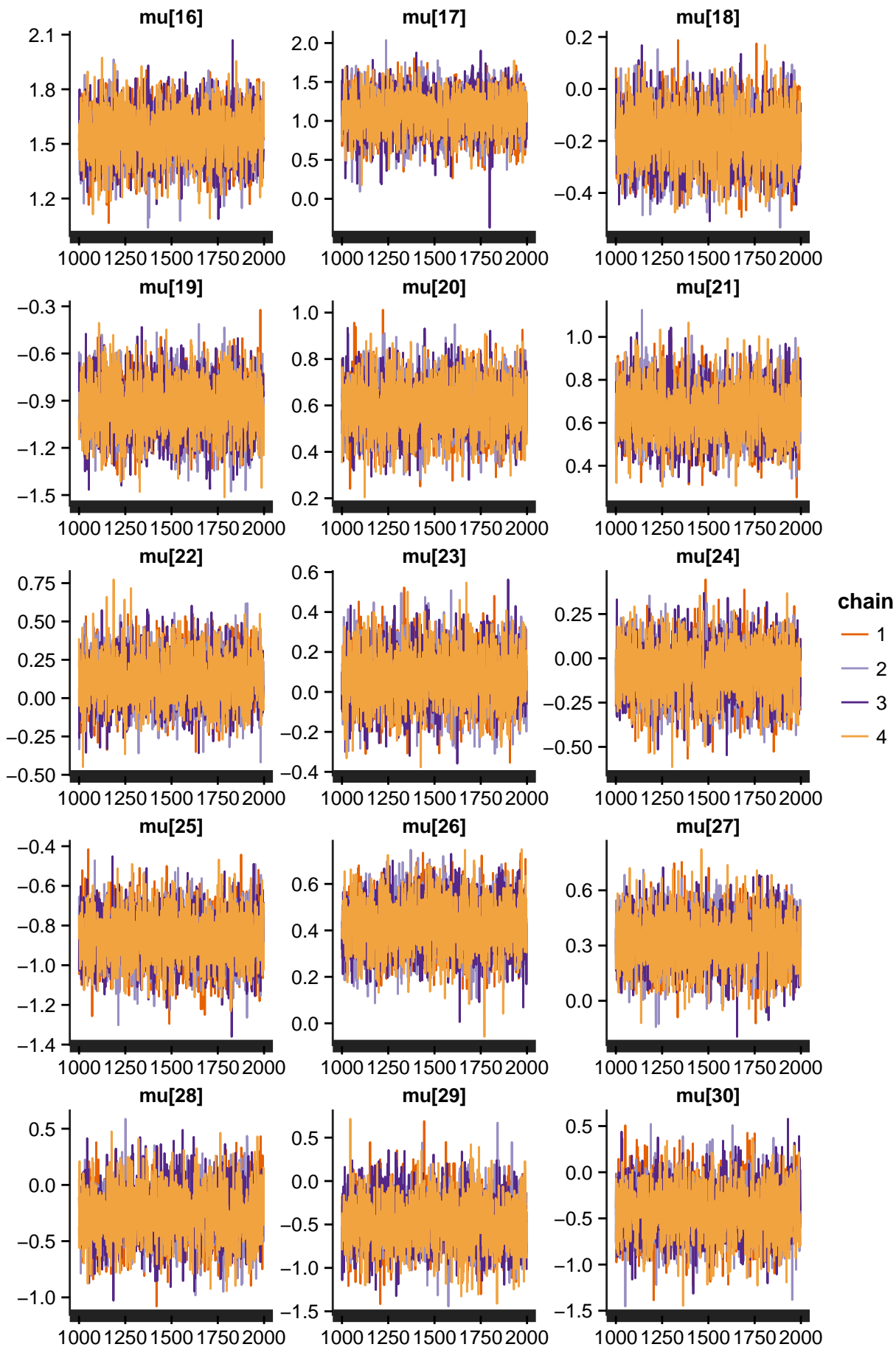

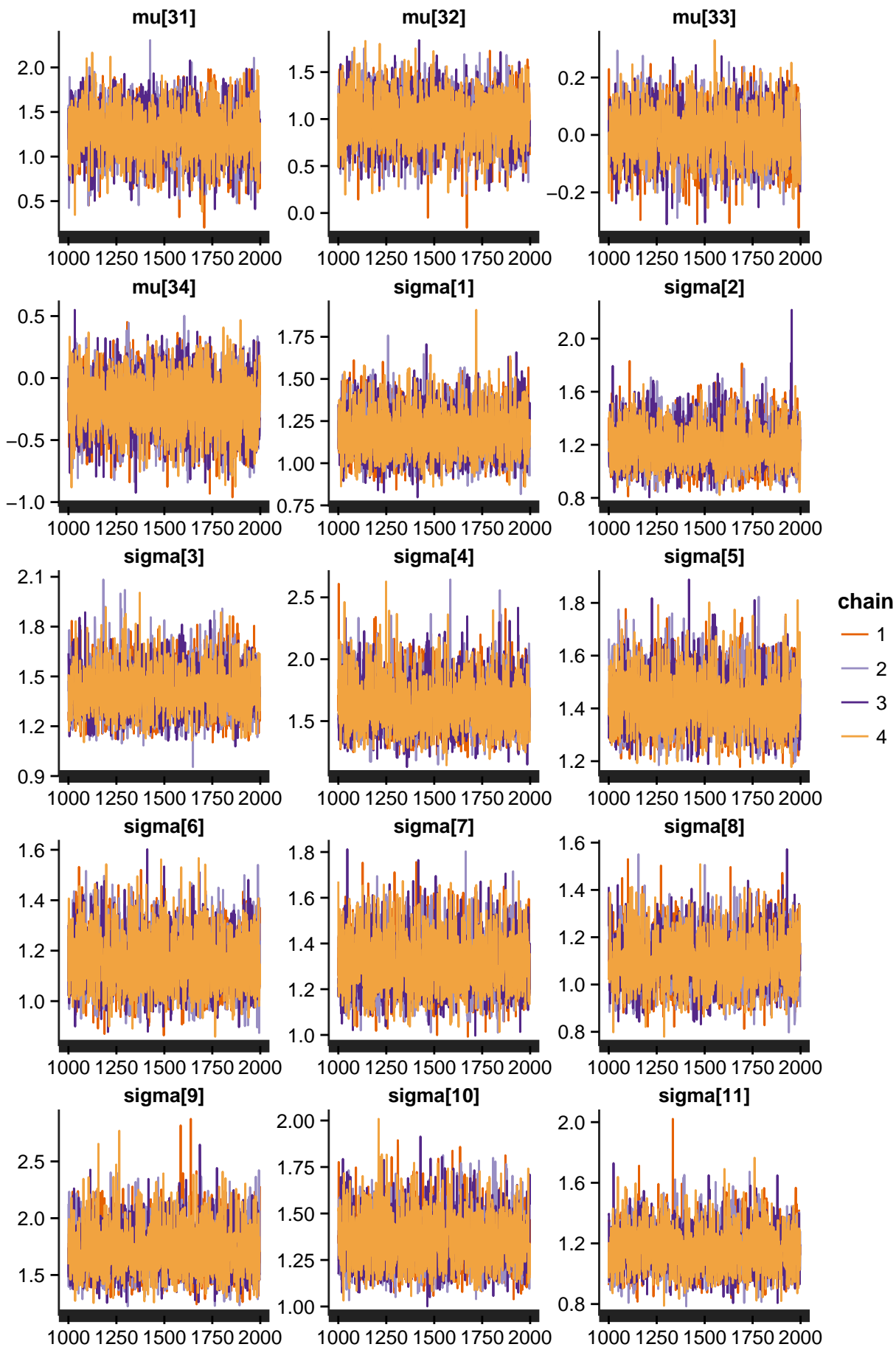

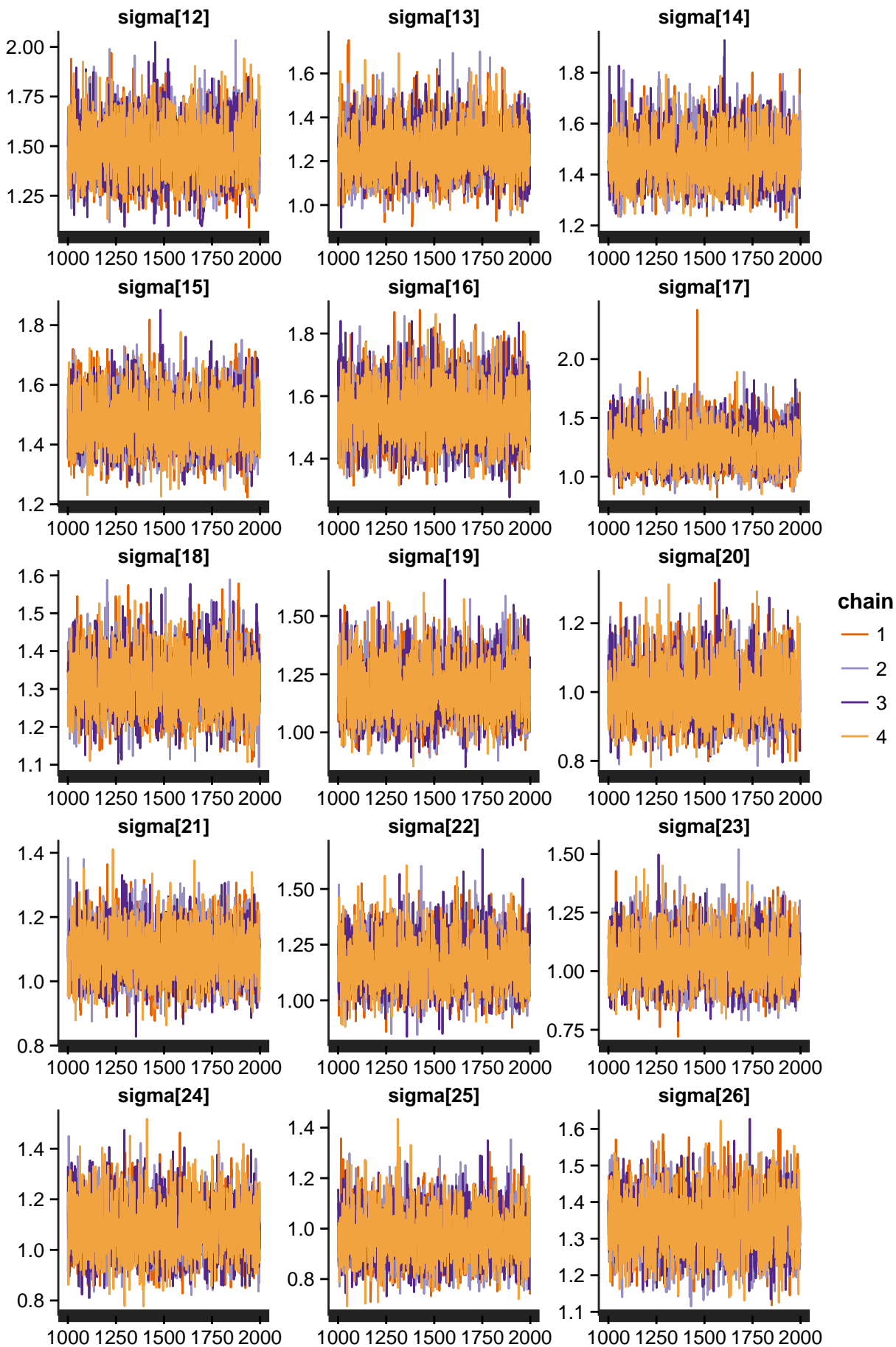

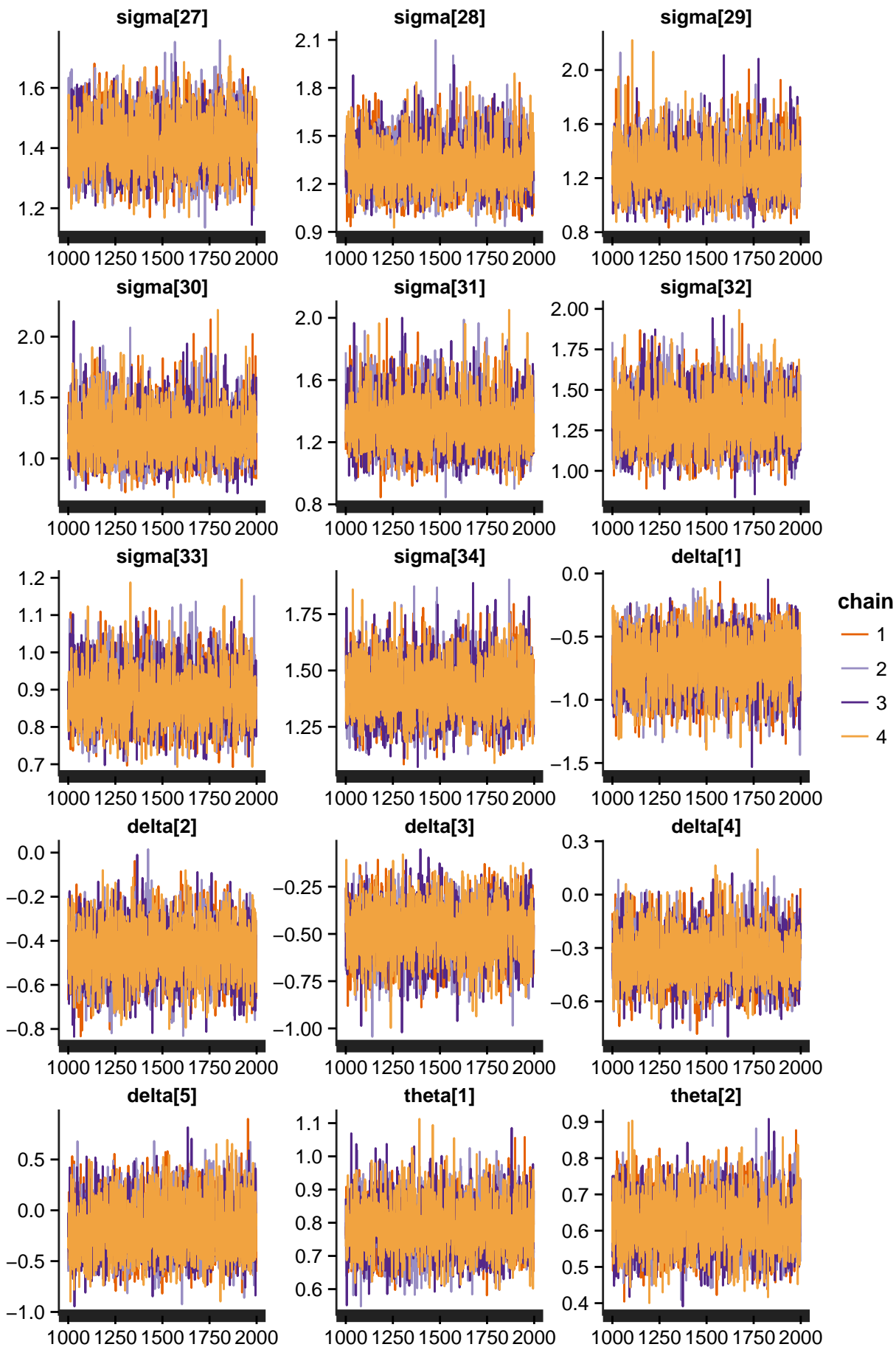

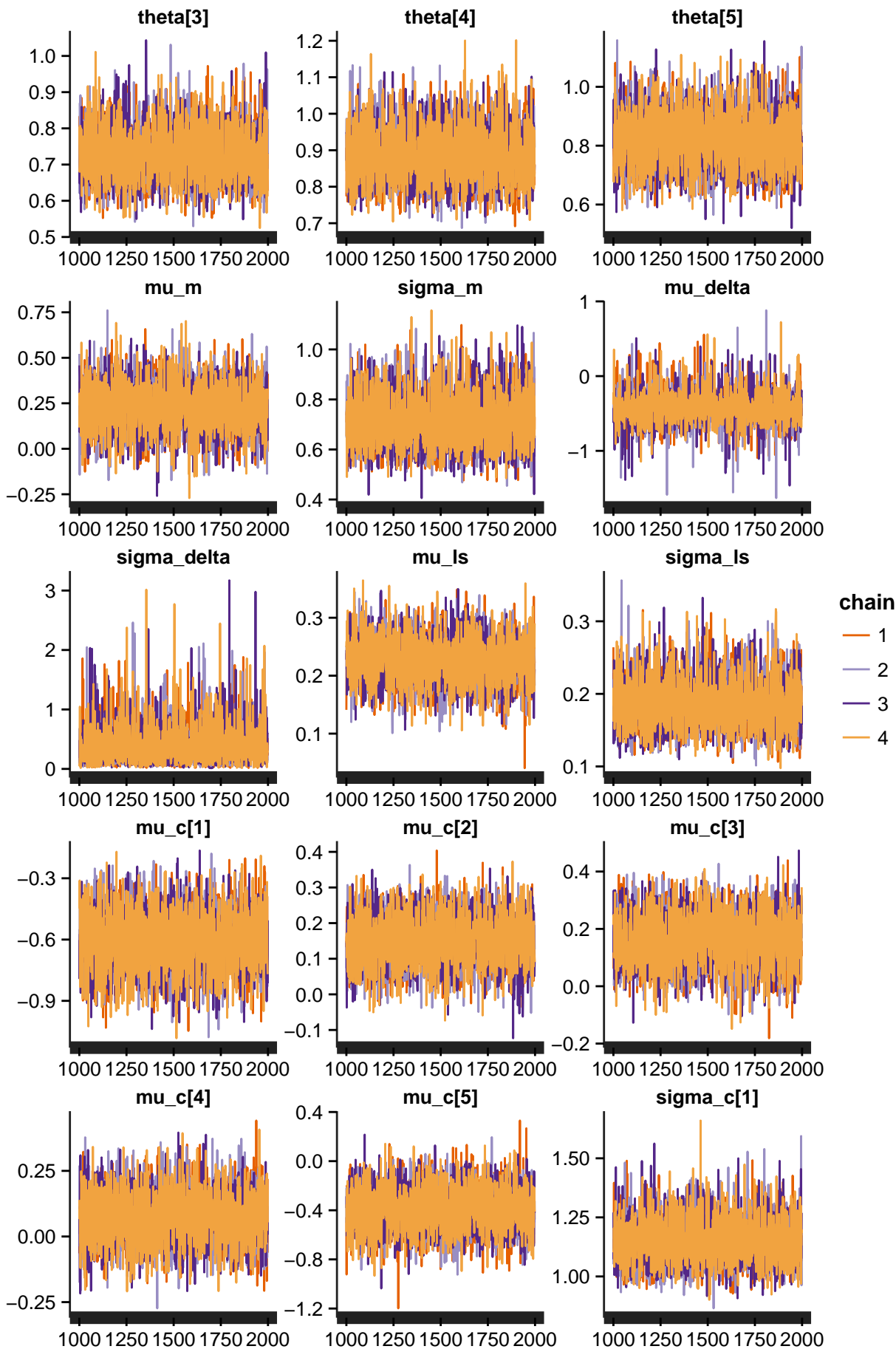

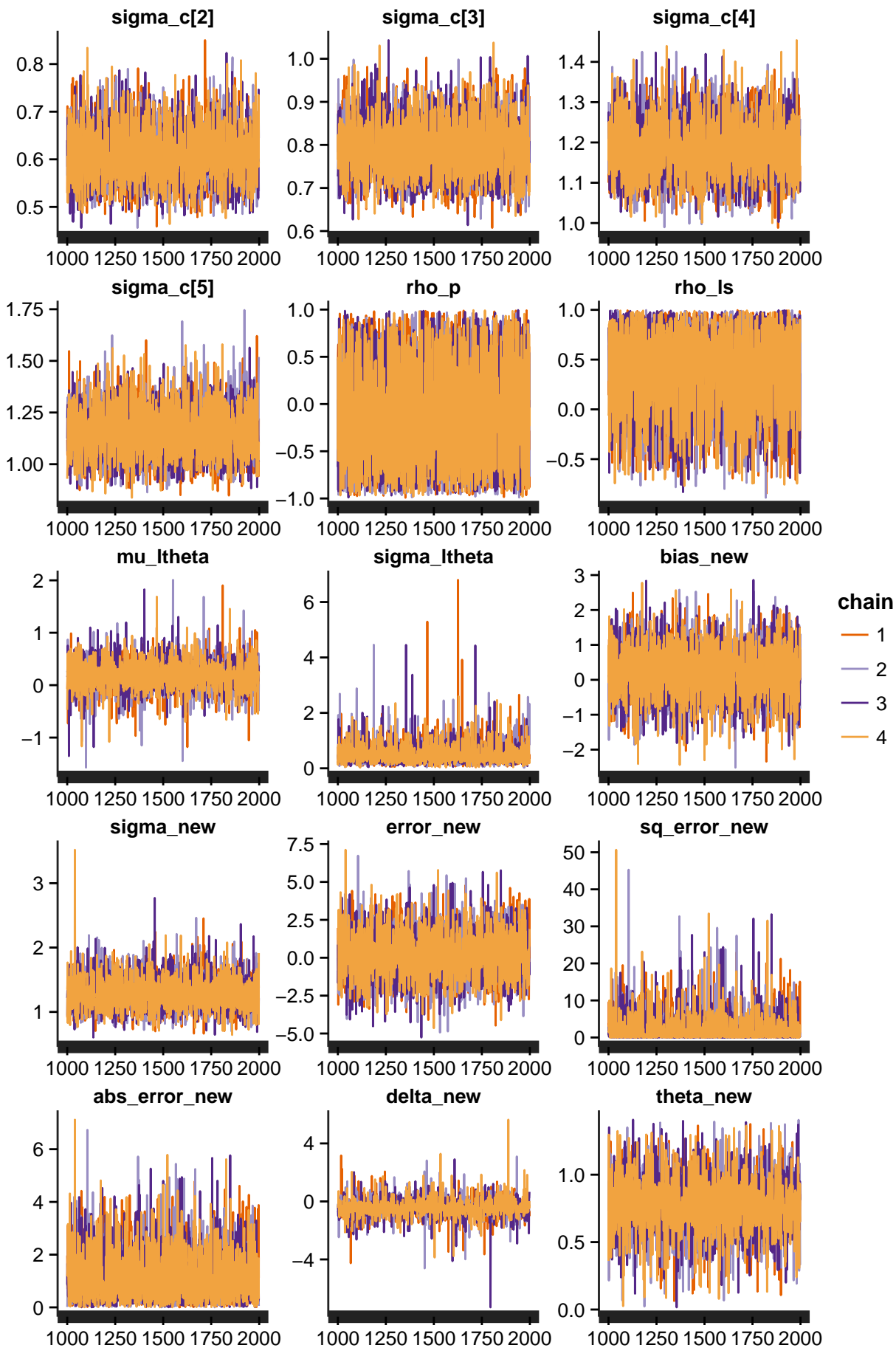

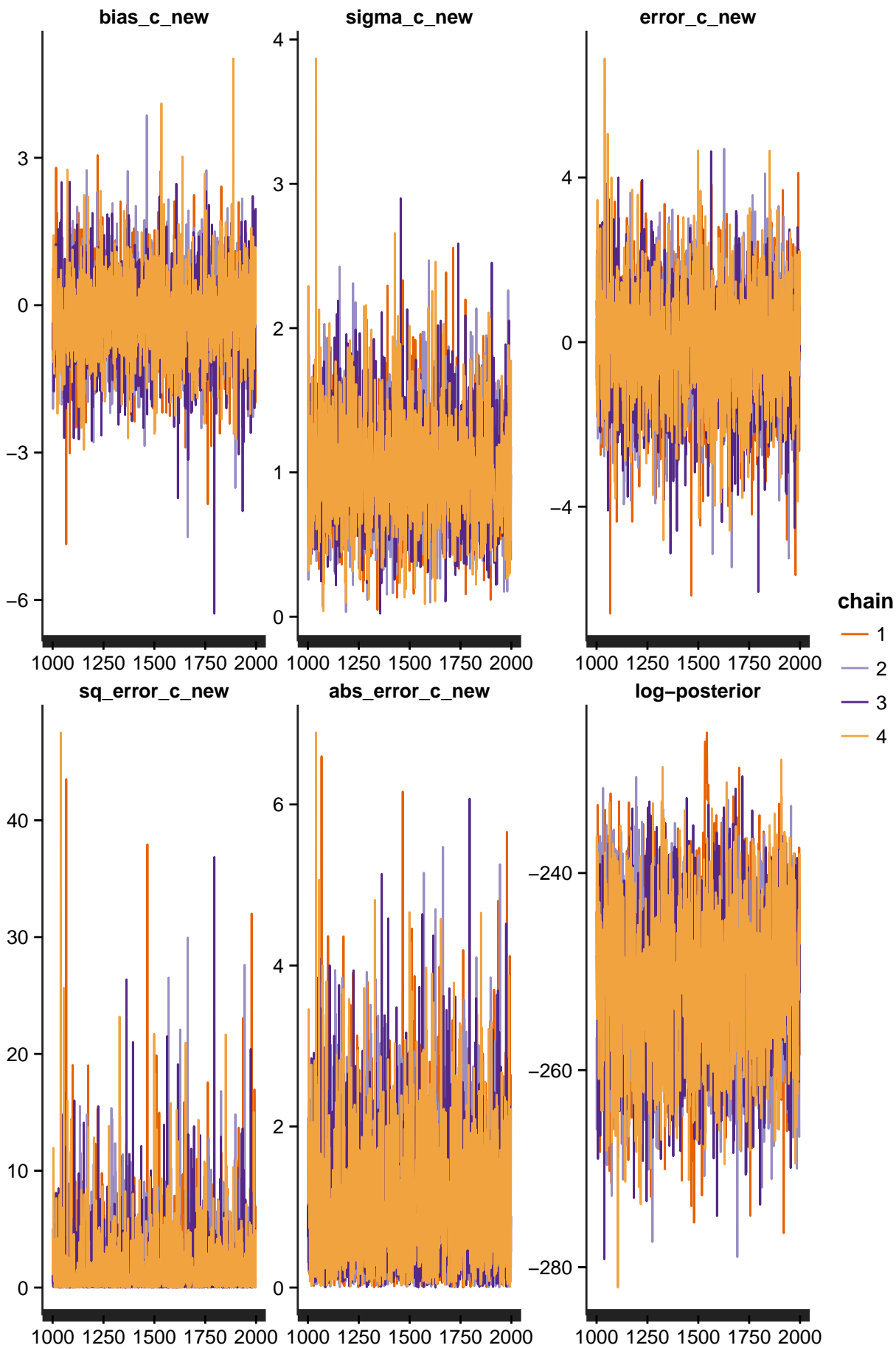

Supplement: Supplementary file 3 — MCMC traces (post-warmup). (PDF 2170.88 kb) [file 12874_2016_107_MOESM3_ESM.pdf]
