# Supplementary material for: Meta-analytic estimation of measurement variability and assessment of its impact on decision-making: the case of perioperative haemoglobin concentration monitoring
Source: BMC Med Res Methodol. 2016 Jan 19;16:7. doi: 10.1186/s12874-016-0107-5 (PMC4717612; doi:10.1186/s12874-016-0107-5)
Supplement: Supplementary file 4 — Summary of the posterior distribution of all parameters in the model. (PDF 41.3 kb) [file 12874_2016_107_MOESM4_ESM.pdf]

Table 1: Full results of MCMC fitting of the model: series-level parameters, SpHb

	mean	se_mean	sd	2.5%	25%	50%	75%	97.5%	n_eff	Rhat
mu[1]	-0.27	0.00	0.20	-0.65	-0.39	-0.27	-0.13	0.13	1608	1.00
mu[2]	-0.25	0.01	0.21	-0.69	-0.39	-0.25	-0.11	0.17	1189	1.00
mu[3]	0.03	0.00	0.22	-0.42	-0.11	0.03	0.18	0.44	2000	1.00
mu[4]	0.29	0.01	0.36	-0.40	0.05	0.29	0.52	0.97	2000	1.00
mu[5]	0.48	0.00	0.15	0.17	0.39	0.49	0.58	0.77	1018	1.00
mu[6]	1.15	0.00	0.15	0.85	1.05	1.16	1.26	1.45	2000	1.00
mu[7]	0.11	0.00	0.18	-0.25	-0.00	0.11	0.23	0.46	2000	1.00
mu[8]	1.35	0.00	0.14	1.06	1.26	1.36	1.45	1.63	1986	1.00
mu[9]	-0.83	0.01	0.42	-1.64	-1.10	-0.83	-0.57	0.07	1759	1.00
mu[10]	0.84	0.00	0.20	0.43	0.70	0.84	0.97	1.22	2000	1.00
mu[11]	-0.17	0.00	0.19	-0.54	-0.30	-0.16	-0.04	0.21	1709	1.00
mu[12]	-0.06	0.00	0.22	-0.50	-0.21	-0.06	0.08	0.37	2000	1.00
mu[13]	0.95	0.00	0.17	0.62	0.84	0.96	1.06	1.27	1830	1.00
mu[14]	0.08	0.00	0.16	-0.21	-0.03	0.08	0.19	0.41	1419	1.01
mu[15]	0.59	0.00	0.13	0.34	0.50	0.59	0.67	0.83	1666	1.00
mu[16]	1.55	0.00	0.13	1.29	1.46	1.55	1.64	1.80	1833	1.00
mu[17]	1.09	0.01	0.25	0.59	0.93	1.08	1.25	1.56	1155	1.00
mu[18]	-0.19	0.00	0.10	-0.39	-0.27	-0.19	-0.12	0.01	1693	1.00
mu[19]	-0.94	0.00	0.16	-1.29	-1.05	-0.94	-0.83	-0.63	1432	1.00
mu[20]	0.59	0.00	0.10	0.39	0.52	0.59	0.66	0.80	2000	1.00
mu[21]	0.65	0.00	0.11	0.43	0.58	0.65	0.73	0.88	1625	1.00
mu[22]	0.13	0.00	0.15	-0.17	0.03	0.13	0.23	0.44	1722	1.00
mu[23]	0.08	0.00	0.13	-0.18	-0.01	0.08	0.16	0.34	1600	1.00
mu[24]	-0.08	0.00	0.14	-0.36	-0.17	-0.08	0.02	0.20	1878	1.00
mu[25]	-0.86	0.00	0.12	-1.11	-0.94	-0.86	-0.78	-0.62	1974	1.00
mu[26]	0.42	0.00	0.11	0.22	0.36	0.43	0.49	0.62	1917	1.00
mu[27]	0.33	0.00	0.13	0.07	0.25	0.33	0.41	0.58	1844	1.00
mu[28]	-0.26	0.01	0.22	-0.69	-0.40	-0.25	-0.11	0.21	1285	1.00
mu[29]	-0.47	0.01	0.27	-1.02	-0.65	-0.46	-0.30	0.08	1933	1.00
mu[30]	-0.41	0.01	0.26	-0.93	-0.58	-0.41	-0.25	0.13	1991	1.00
mu[31]	1.25	0.01	0.27	0.67	1.08	1.26	1.43	1.77	968	1.00
mu[32]	0.98	0.01	0.24	0.49	0.82	0.99	1.14	1.45	2000	1.00
mu[33]	0.00	0.00	0.09	-0.17	-0.06	0.00	0.06	0.17	1574	1.00
mu[34]	-0.20	0.01	0.23	-0.67	-0.35	-0.19	-0.04	0.21	2000	1.00
sigma[1]	1.18	0.00	0.13	0.95	1.09	1.17	1.26	1.45	1906	1.00
sigma[2]	1.19	0.00	0.15	0.93	1.09	1.18	1.29	1.52	1769	1.00
sigma[3]	1.41	0.00	0.13	1.18	1.32	1.40	1.50	1.70	1567	1.00
sigma[4]	1.63	0.01	0.20	1.30	1.49	1.61	1.76	2.06	1087	1.00
sigma[5]	1.43	0.00	0.10	1.25	1.36	1.43	1.49	1.64	1390	1.01
sigma[6]	1.15	0.00	0.11	0.97	1.07	1.14	1.21	1.37	1267	1.00
sigma[7]	1.31	0.00	0.12	1.11	1.22	1.30	1.39	1.57	1608	1.00
sigma[8]	1.10	0.00	0.11	0.91	1.02	1.09	1.16	1.33	1639	1.00
sigma[9]	1.72	0.00	0.21	1.37	1.57	1.71	1.85	2.18	1945	1.00
sigma[10]	1.36	0.00	0.13	1.13	1.27	1.35	1.44	1.67	1261	1.00
sigma[11]	1.15	0.00	0.14	0.92	1.06	1.14	1.23	1.45	1413	1.00
sigma[12]	1.47	0.00	0.14	1.21	1.38	1.46	1.56	1.76	1114	1.00
sigma[13]	1.25	0.00	0.11	1.05	1.17	1.25	1.33	1.49	2000	1.00
sigma[14]	1.47	0.00	0.09	1.30	1.40	1.46	1.52	1.67	1547	1.00
sigma[15]	1.48	0.00	0.08	1.32	1.42	1.47	1.53	1.65	1643	1.00
sigma[16]	1.55	0.00	0.09	1.38	1.48	1.54	1.60	1.74	1908	1.00
sigma[17]	1.25	0.00	0.16	0.97	1.14	1.24	1.35	1.59	1518	1.00
sigma[18]	1.31	0.00	0.07	1.18	1.26	1.31	1.36	1.47	2000	1.00
sigma[19]	1.18	0.00	0.11	0.99	1.11	1.17	1.25	1.42	2000	1.00
sigma[20]	1.00	0.00	0.08	0.86	0.94	0.99	1.05	1.17	1439	1.00
sigma[21]	1.09	0.00	0.08	0.94	1.03	1.09	1.14	1.24	1924	1.00
sigma[22]	1.16	0.00	0.11	0.97	1.09	1.16	1.23	1.40	1700	1.00
sigma[23]	1.05	0.00	0.10	0.87	0.98	1.04	1.12	1.27	1427	1.00
sigma[24]	1.08	0.00	0.10	0.90	1.01	1.08	1.14	1.29	2000	1.00
sigma[25]	0.97	0.00	0.09	0.80	0.90	0.96	1.03	1.17	1396	1.00
sigma[26]	1.34	0.00	0.08	1.20	1.28	1.33	1.38	1.50	1476	1.00
sigma[27]	1.42	0.00	0.09	1.26	1.36	1.41	1.47	1.59	1459	1.00
sigma[28]	1.30	0.00	0.15	1.06	1.20	1.29	1.39	1.63	1208	1.01
sigma[29]	1.25	0.00	0.17	0.96	1.14	1.24	1.35	1.64	1597	1.00
sigma[30]	1.20	0.00	0.20	0.88	1.07	1.19	1.32	1.64	1748	1.00
sigma[31]	1.30	0.00	0.16	1.04	1.19	1.29	1.40	1.67	2000	1.00
sigma[32]	1.31	0.00	0.15	1.05	1.21	1.30	1.41	1.65	1440	1.00
sigma[33]	0.88	0.00	0.07	0.75	0.83	0.88	0.93	1.02	1696	1.00
sigma[34]	1.41	0.00	0.11	1.21	1.33	1.40	1.49	1.66	1810	1.00

Table 2: Full results of MCMC fitting of the model: series-level parameters, cSpHb

	mean	se_mean	sd	2.5%	25%	50%	75%	97.5%	n_eff	Rhat
delta[1]	-0.69	0.01	0.22	-1.15	-0.84	-0.68	-0.53	-0.33	1257	1.00
delta[2]	-0.45	0.00	0.12	-0.68	-0.52	-0.45	-0.37	-0.22	1960	1.00
delta[3]	-0.49	0.00	0.13	-0.76	-0.58	-0.48	-0.40	-0.23	1619	1.00
delta[4]	-0.35	0.00	0.14	-0.60	-0.44	-0.36	-0.26	-0.07	1485	1.00
delta[5]	-0.19	0.01	0.28	-0.62	-0.41	-0.23	0.00	0.40	1627	1.00
mu_c[1]	-0.61	0.00	0.14	-0.89	-0.70	-0.61	-0.51	-0.33	1835	1.00
mu_c[2]	0.15	0.00	0.07	0.02	0.10	0.15	0.19	0.28	1699	1.00
mu_c[3]	0.16	0.00	0.09	-0.02	0.10	0.16	0.22	0.33	1687	1.00
mu_c[4]	0.07	0.00	0.10	-0.12	0.01	0.07	0.14	0.27	1326	1.00
mu_c[5]	-0.39	0.00	0.17	-0.71	-0.51	-0.40	-0.28	-0.04	2000	1.00
theta[1]	0.79	0.00	0.07	0.65	0.74	0.78	0.83	0.94	1961	1.00
theta[2]	0.62	0.00	0.07	0.48	0.56	0.61	0.66	0.77	1386	1.00
theta[3]	0.73	0.00	0.07	0.61	0.68	0.73	0.78	0.87	1888	1.00
theta[4]	0.89	0.00	0.07	0.76	0.84	0.89	0.93	1.04	1319	1.00
theta[5]	0.82	0.00	0.09	0.65	0.75	0.81	0.87	1.00	1126	1.00
sigma_c[1]	1.15	0.00	0.09	0.99	1.08	1.14	1.21	1.35	1975	1.00
sigma_c[2]	0.61	0.00	0.06	0.51	0.57	0.61	0.65	0.72	1742	1.00
sigma_c[3]	0.79	0.00	0.06	0.68	0.75	0.79	0.83	0.93	2000	1.00
sigma_c[4]	1.18	0.00	0.07	1.06	1.13	1.18	1.23	1.33	1806	1.00
sigma_c[5]	1.15	0.00	0.12	0.94	1.06	1.13	1.22	1.40	2000	1.00

Table 3: Full results of MCMC fitting of the model: population parameters and derived quantities.

	mean	se_mean	sd	2.5%	25%	50%	75%	97.5%	n_eff	Rhat
mu_m	0.23	0.00	0.12	-0.02	0.15	0.23	0.31	0.46	2000	1.00
sigma_m	0.71	0.00	0.10	0.54	0.63	0.70	0.77	0.94	1215	1.00
mu_delta	-0.42	0.00	0.20	-0.83	-0.53	-0.43	-0.33	0.02	1677	1.00
sigma_delta	0.39	0.01	0.33	0.03	0.18	0.31	0.51	1.28	1338	1.00
mu_ls	0.23	0.00	0.04	0.15	0.20	0.23	0.25	0.30	941	1.00
sigma_ls	0.18	0.00	0.03	0.13	0.16	0.18	0.20	0.26	1626	1.00
rho_p	-0.13	0.01	0.56	-0.92	-0.62	-0.22	0.32	0.92	1418	1.00
rho_ls	0.39	0.01	0.42	-0.52	0.11	0.46	0.73	0.97	1792	1.00
mu_ltheta	0.19	0.01	0.26	-0.33	0.04	0.18	0.32	0.73	1913	1.00
sigma_ltheta	0.51	0.01	0.39	0.09	0.28	0.42	0.64	1.43	1869	1.00
bias_new	0.24	0.02	0.73	-1.23	-0.22	0.23	0.75	1.59	1500	1.00
sigma_new	1.28	0.01	0.24	0.86	1.12	1.26	1.42	1.82	1891	1.00
error_new	0.27	0.03	1.47	-2.56	-0.68	0.28	1.20	3.26	2000	1.00
sq_error_new	2.24	0.08	3.54	0.00	0.20	0.95	2.78	11.31	2000	1.00
abs_error_new	1.18	0.02	0.92	0.05	0.45	0.97	1.67	3.36	2000	1.00
delta_new	-0.44	0.01	0.59	-1.55	-0.64	-0.44	-0.24	0.70	1910	1.00
theta_new	0.77	0.00	0.19	0.36	0.66	0.77	0.88	1.17	1936	1.00
bias_c_new	-0.20	0.02	0.87	-1.99	-0.70	-0.18	0.31	1.49	1706	1.00
sigma_c_new	1.00	0.01	0.35	0.39	0.77	0.97	1.18	1.81	1887	1.00
error_c_new	-0.20	0.03	1.35	-2.92	-1.04	-0.20	0.63	2.52	2000	1.00
sq_error_c_new	1.86	0.07	3.16	0.00	0.16	0.73	2.12	10.29	2000	1.00
abs_error_c_new	1.05	0.02	0.87	0.04	0.40	0.86	1.46	3.21	2000	1.00
lp__	-250.40	0.30	7.81	-265.86	-255.61	-250.36	-245.23	-235.78	657	1.01
