# Supplementary material for: Meta-analytic estimation of measurement variability and assessment of its impact on decision-making: the case of perioperative haemoglobin concentration monitoring
Source: BMC Med Res Methodol. 2016 Jan 19;16:7. doi: 10.1186/s12874-016-0107-5 (PMC4717612; doi:10.1186/s12874-016-0107-5)
Supplement: Supplementary file 5 — Boxplots of study-level parameters distributions against the observed data they model. (PDF 27.2 kb) [file 12874_2016_107_MOESM5_ESM.pdf]

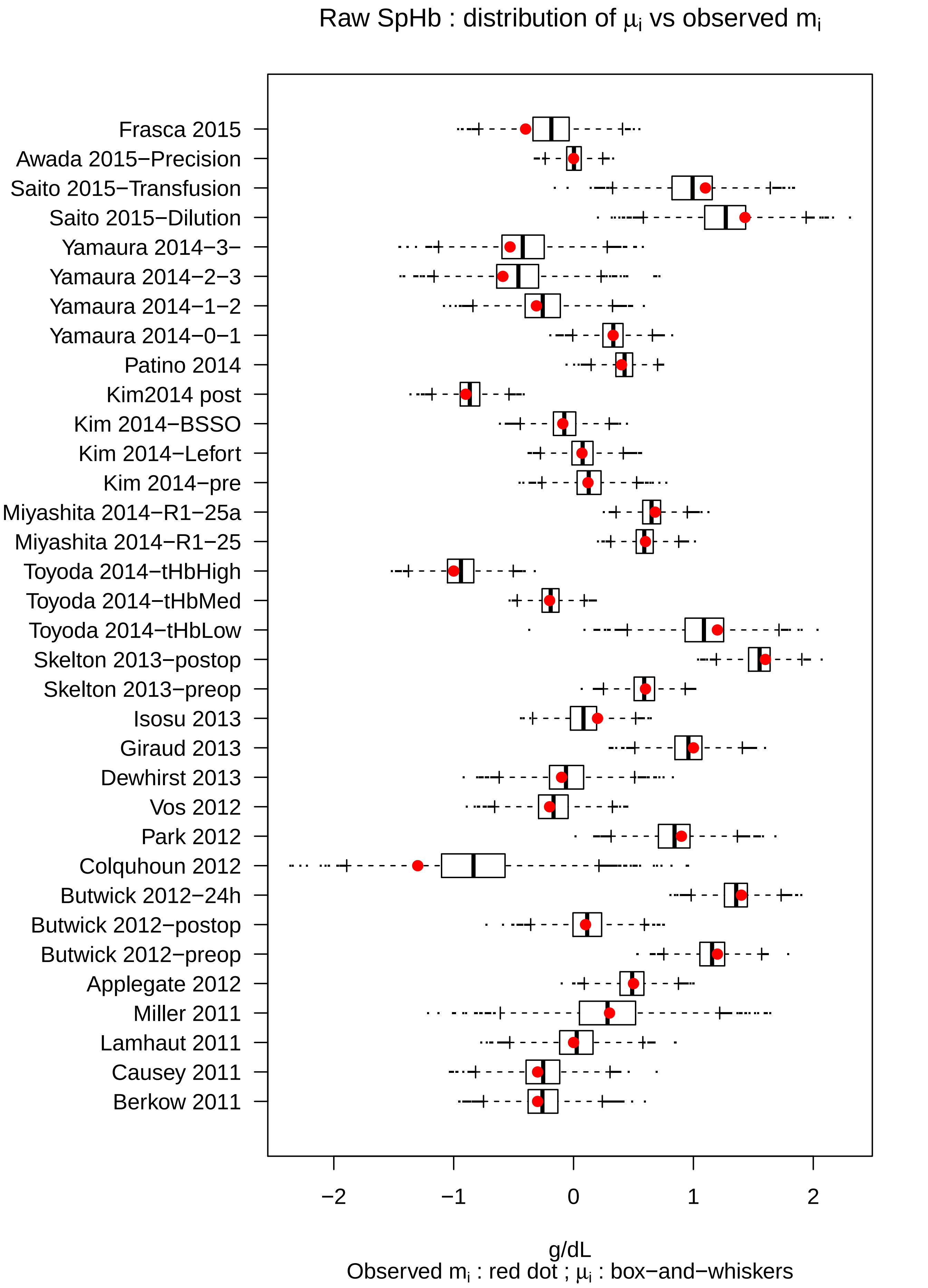

Raw SpHb : distribution of μ_i vs observed m_i
Frasca 2015
Awada 2015−Precision
Saito 2015−Transfusion
Saito 2015−Dilution
Yamaura 2014−3−
Yamaura 2014−2−3
Yamaura 2014−1−2
Yamaura 2014−0−1
Patino 2014
Kim2014 post
Kim 2014−BSSO
Kim 2014−Lefort
Kim 2014−pre
Miyashita 2014−R1−25a
Miyashita 2014−R1−25
Toyoda 2014−tHbHigh
Toyoda 2014−tHbMed
Toyoda 2014−tHbLow
Skelton 2013−postop
Skelton 2013−preop
Isosu 2013
Giraud 2013
Dewhirst 2013
Vos 2012
Park 2012
Colquhoun 2012
Butwick 2012−24h
Butwick 2012−postop
Butwick 2012−preop
Applegate 2012
Miller 2011
Lamhaut 2011
Causey 2011
Berkow 2011
−2
−1
0
1
2
g/dL
Observed m_i : red dot ; μ_i : box−and−whiskers

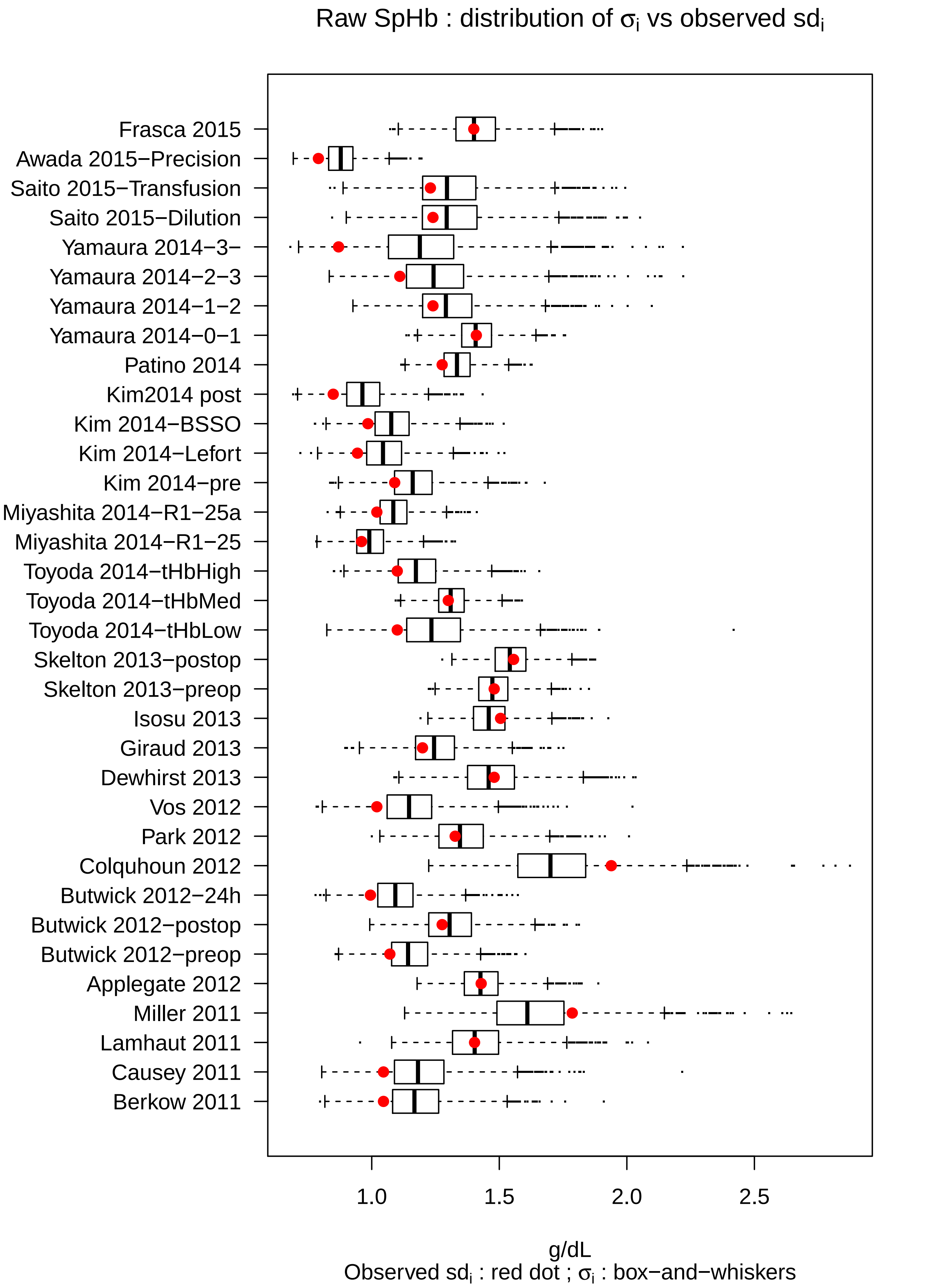

Raw SpHb : distribution of σ_i vs observed sd_i
Frasca 2015
Awada 2015−Precision
Saito 2015−Transfusion
Saito 2015−Dilution
Yamaura 2014−3−
Yamaura 2014−2−3
Yamaura 2014−1−2
Yamaura 2014−0−1
Patino 2014
Kim2014 post
Kim 2014−BSSO
Kim 2014−Lefort
Kim 2014−pre
Miyashita 2014−R1−25a
Miyashita 2014−R1−25
Toyoda 2014−tHbHigh
Toyoda 2014−tHbMed
Toyoda 2014−tHbLow
Skelton 2013−postop
Skelton 2013−preop
Isosu 2013
Giraud 2013
Dewhirst 2013
Vos 2012
Park 2012
Colquhoun 2012
Butwick 2012−24h
Butwick 2012−postop
Butwick 2012−preop
Applegate 2012
Miller 2011
Lamhaut 2011
Causey 2011
Berkow 2011
1.0
1.5
2.0
2.5
g/dL
Observed sd_i : red dot ; σ_i : box−and−whiskers

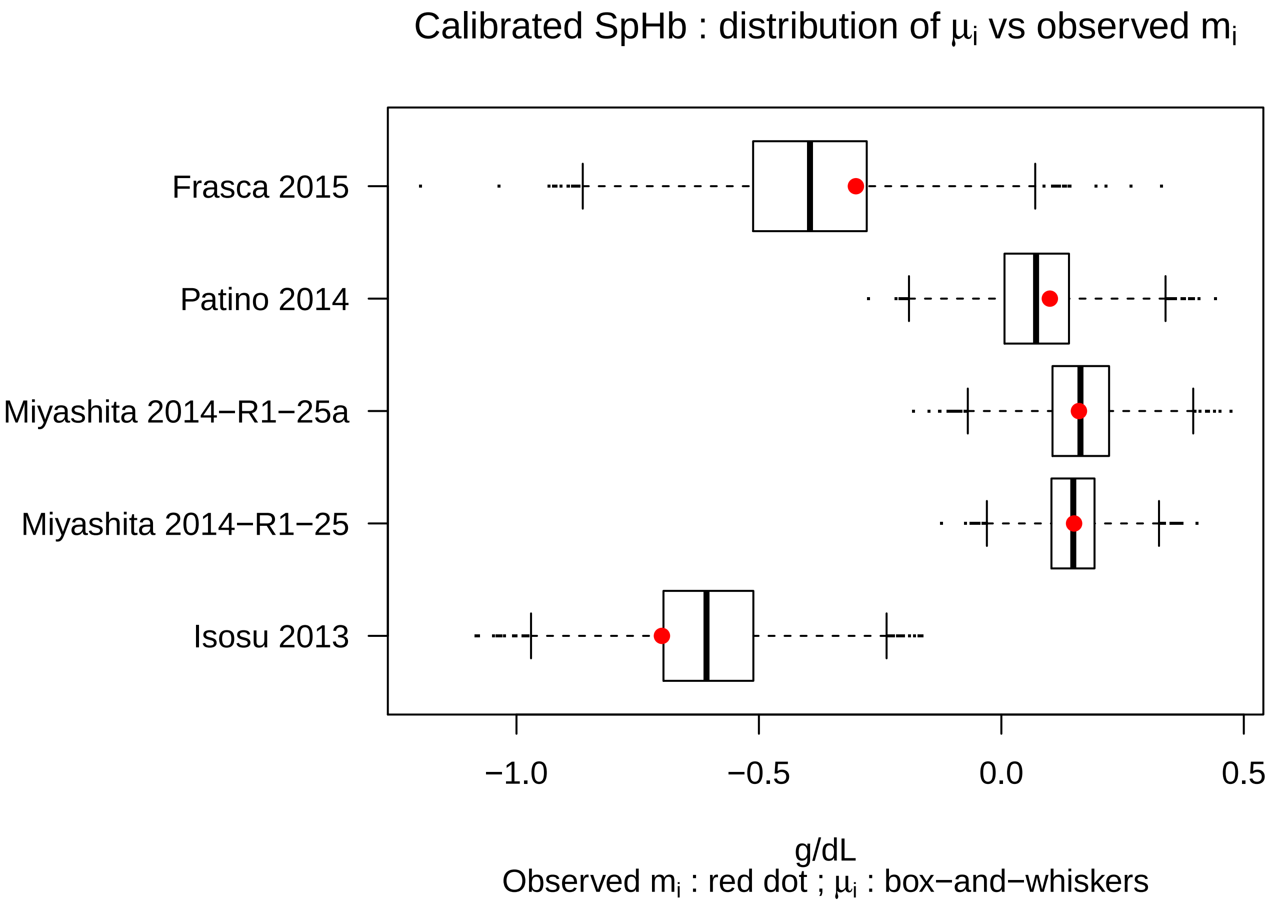

Calibrated SpHb : distribution of μ_i vs observed m_i
Frasca 2015
Patino 2014
Miyashita 2014−R1−25a
Miyashita 2014−R1−25
Isosu 2013
−1.0
−0.5
0.0
0.5
g/dL
Observed m_i : red dot ; μ_i : box−and−whiskers

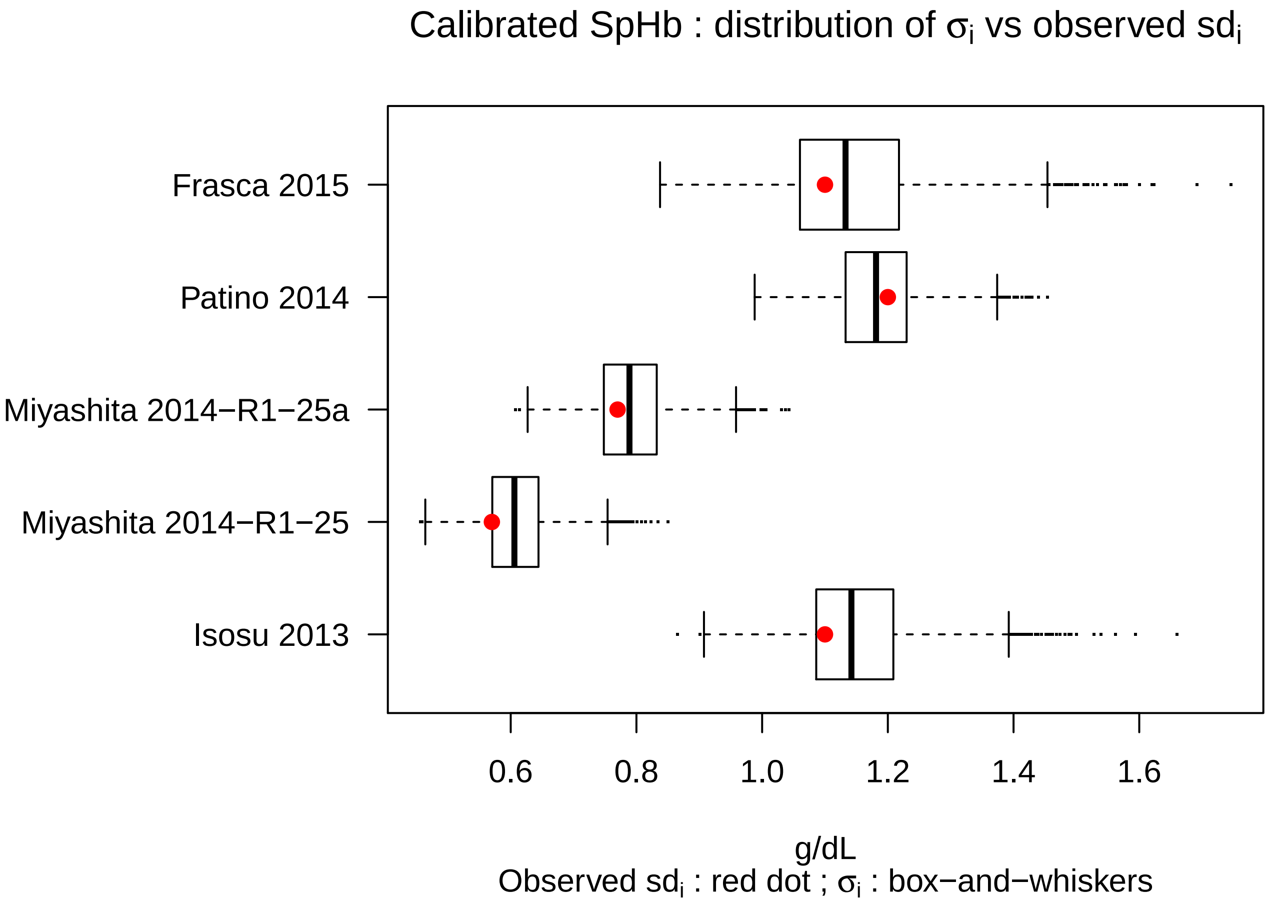

Calibrated SpHb : distribution of σ_i vs observed sd_i
Frasca 2015
Patino 2014
Miyashita 2014−R1−25a
Miyashita 2014−R1−25
Isosu 2013
0.6
0.8
1.0
1.2
1.4
1.6
g/dL
Observed sd_i : red dot ; σ_i : box−and−whiskers
